# Parathyroid hormone receptor signalling in osterix-expressing mesenchymal progenitors is essential for tooth root formation

**DOI:** 10.1038/ncomms11277

**Published:** 2016-04-12

**Authors:** Wanida Ono, Naoko Sakagami, Shigeki Nishimori, Noriaki Ono, Henry M. Kronenberg

**Affiliations:** 1 Endocrine Unit, Massachusetts General Hospital and Harvard Medical School, Boston, Massachusetts 02114, USA; 2 Department of Orthodontics and Pediatric Dentistry, University of Michigan School of Dentistry, Ann Arbor, Michigan 48109, USA

## Abstract

Dental root formation is a dynamic process in which mesenchymal cells migrate toward the site of the future root, differentiate and secrete dentin and cementum. However, the identities of dental mesenchymal progenitors are largely unknown. Here we show that cells expressing osterix are mesenchymal progenitors contributing to all relevant cell types during morphogenesis. The majority of cells expressing parathyroid hormone-related peptide (*PTHrP*) are in the dental follicle and on the root surface, and deletion of its receptor (PPR) in these progenitors leads to failure of eruption and significantly truncated roots lacking periodontal ligaments. The PPR-deficient progenitors exhibit accelerated cementoblast differentiation with upregulation of nuclear factor I/C (*Nfic*). Deletion of histone deacetylase-4 (HDAC4) partially recapitulates the PPR deletion root phenotype. These findings indicate that PPR signalling in dental mesenchymal progenitors is essential for tooth root formation, underscoring importance of the PTHrP–PPR system during root morphogenesis and tooth eruption.

## Abstract (web summary)

How the parathyroid hormone-related protein receptor (PPR) and its ligand act in root formation and tooth eruption is unclear. Here, the authors identify osterix-expressing dental mesenchymal cells as progenitors for root formation and that PPR signalling mediates their differentiation and tooth eruption.

## Graphical abstract

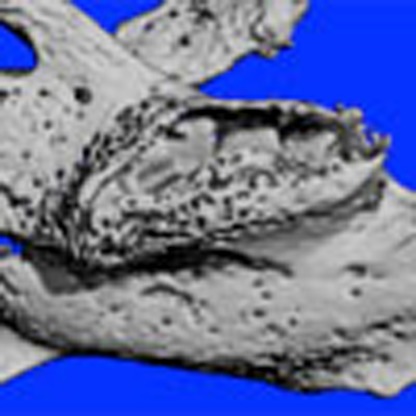

Tooth morphogenesis, characterized by the two distinct stages of crown and root formation, is a prime example of organogenesis involving sequential steps of reciprocal epithelial–mesenchymal interactions^1^. During crown formation, the invaginated dental epithelial cells differentiate into ameloblasts that form the enamel, and then their adjacent dental papilla mesenchymal cells differentiate into odontoblasts that form the dentin. Subsequent root formation is initiated by formation of a bilayered tissue termed as Hertwig's epithelial root sheath (HERS)^2^. The epithelial root sheath continues to grow downward to shape the future root of the tooth, and dental papilla and follicle mesenchymal cells in its vicinity differentiate into matrix-producing odontoblasts and cementoblasts to form dentin (inside) and cementum (outside), respectively (Fig. 1a). The dental root is a critical component of the tooth, anchored to surrounding alveolar bones by the periodontal ligament (PDL). Both human and rodent molars have multiple roots and are formed through identical developmental sequences, and formation of the tooth root and its surrounding structure, including the PDL and alveolar bone, is considered to be important for tooth eruption^3^.

Several lines of evidence suggest that a genetic programme similar to that used during osteoblast differentiation, such as transcription factors Runx2 and osterix (Osx), governs the process of dental mesenchymal cell differentiation during root morphogenesis. Runx2- and Osx-deficient mice exhibit failure of tooth mineralization, although the role of these transcription factors in root formation is unknown because the knockout mice die before the onset of root formation^4^,^5^. Osx is expressed in odontoblasts, alveolar bone osteoblasts and dental follicle cells during tooth development^6^, and regulates cementum formation^7^. In the early foetal perichondrium of endochondral bones, Osx-expressing osteoblast precursors translocate to bone marrow and become osteoblasts and stromal cells inside the developing bone, while mature osteoblasts already expressing type I collagen (Col1) contributed only to osteoblasts in the bone collar^8^. Whether cells expressing Osx behave as odontoblast and cementoblast progenitors during dental root development is unknown.

The parathyroid hormone-related protein (PTHrP), a locally acting autocrine/paracrine ligand, and its receptor, the PTH/PTHrP receptor (PPR), mediate a number of important biological actions such as endochondral bone development^9^,^10^. PTHrP binds to the PPR and activates multiple heterotrimeric G proteins and triggers the intracellular signalling pathways. In chondrocytes, the PTHrP–PPR system suppresses hypertrophy through a protein kinase A (PKA) pathway-activated nuclear localization of histone deacetylace-4 (HDAC4) that inhibits MEF2 function^11^. In addition, the Gsα signal downstream of the PPR in cells of the osteoblast lineage facilitates the commitment of mesenchymal progenitors to the osteoblast lineage and restrains the differentiation of committed osteoblasts^12^. Previous studies indicate that the PTHrP–PPR system also plays important roles in tooth eruption. In humans, loss-of-function mutations of the *PPR* segregate with familial non-syndromic primary failure of tooth eruption^13^,^14^. The PTHrP-deficient mice whose lethal skeletal defect is rescued by expressing PTHrP in chondrocytes show failure of tooth eruption, and rescuing the mice further by expressing PTHrP in epithelial cells corrects tooth eruption^15^. Furthermore, PTHrP regulates extracellular matrix gene expression in cementoblastic cell lines and inhibits mineralization *in vitro*^16^. However, the underlying mechanisms whereby the PTHrP–PPR system regulates root formation and tooth eruption *in vivo* are largely unknown.

In this study, we hypothesize that the PPR signalling regulates proliferation and differentiation of osterix-expressing progenitors, and plays an essential role in dental root formation and tooth eruption. Our data reveal that the PPR signalling in dental mesenchymal progenitors is essential for orchestrated differentiation of cementoblasts and PDL cells, underscoring the importance of the PTHrP–PPR system in dental root formation and tooth eruption.

## Results

### Progenitors of tooth root-forming cells express osterix

**Figure 2:**
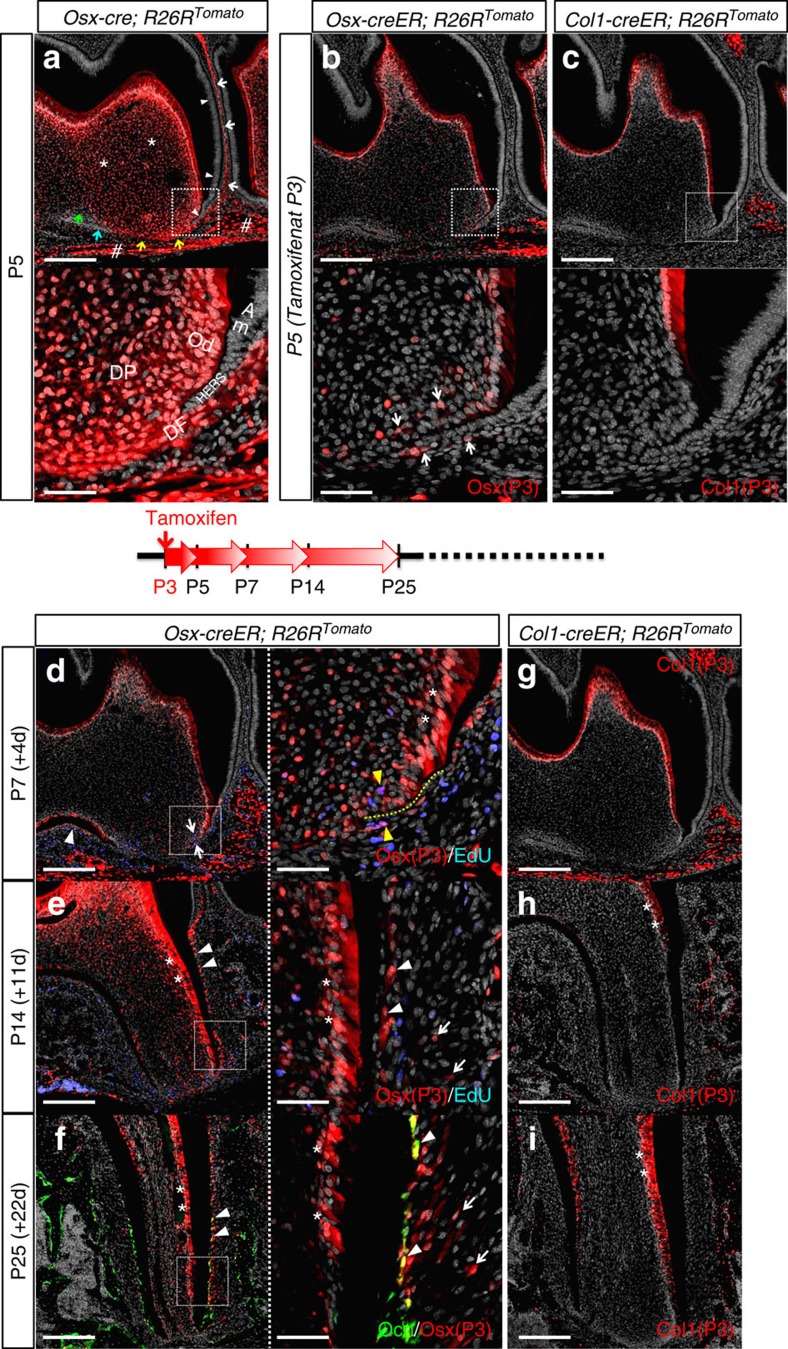
Mesenchymal progenitors of dental root-forming cells express osterix (Osx). (**a**–**c**) P5 mandibular first molar (M1) sections of (**a**) *Osx-cre*; *R26R*^Tomato^, (**b**) *Osx-creER*; *R26R*^Tomato^ (tamoxifen at P3) and (**c**) *Col1-creER*; *R26R*^Tomato^ (tamoxifen at P3) mice were stained for nuclei. Upper panels: confocal images of the distal root, lower panels: magnified views of the dotted areas. Red: tdTomato, grey: DAPI. In (**a**), white arrows: dental follicle, white arrowheads: ameloblasts and epithelial root sheath, asterisks: dental papilla, sharps: alveolar bone, yellow/blue arrows: Tomato^−^ dental follicle, green arrows: Tomato^−^ dental papilla. DP: dental papilla, DF: dental follicle, Od: odontoblasts, Am: ameloblasts. In (**b**), arrows: dental papilla and follicle cells surrounding HERS. Scale bars: 200 μm (upper panel) and 50 μm (lower panel). (**d**,**e**) *Osx-creER*; *R26R*^Tomato^ mice received 0.1 mg tamoxifen at P3 and were analyzed at P7 (**d**) and P14 (**e**). EdU was administered twice (6 and 3 h) prior to analysis. M1 sections were stained for nuclei and EdU. Left panels: confocal images of the distal root, right panels: magnified views of the dotted areas. Red: tdTomato, grey: DAPI, blue: EdU-Alexa647. In (**d**), white arrows: red cells surrounding HERS, white arrowhead: red cells in the interradicular area, yellow dotted line: HERS, yellow arrowheads: EdU^+^ red cells. In (**e**), arrowheads: cementoblasts, asterisks: odontoblasts, arrows: PDL cells. Scale bars: 200 μm (left panel) and 50 μm (right panel). (**f**) *Oc-GFP; Osx-creER*; *R26R*^Tomato^ mice received 0.1 mg tamoxifen at P3 and were analyzed at P25. M1 sections were stained for nuclei. Left panel: a confocal image of the distal root, right panel: a magnified view of the dotted area. Green: EGFP, red: tdTomato, grey: DAPI. Arrowheads: *Oc*-GFP^+^ cementoblasts, asterisks: odontoblasts, arrows: PDL cells. Scale bars: 200 μm (left panel) and 50 μm (right panel). (**g**–**i**) *Col1-creER*; *R26R*^Tomato^ mice received tamoxifen at P3 and were analyzed at P7 (**g**), P14 (**h**) and P25 (**i**). M1 sections were stained for nuclei. Red: tdTomato, grey: DAPI. Asterisks: odontoblasts. Scale bars: 200 μm.

To understand how osterix-expressing cells participate in dental root morphogenesis of murine molars, we first mapped cell fates using a constitutively active *Osx-cre* and an *R26R*-tomato reporter system. In this system, cells expressing Osx and their descendants become permanently red. The mandibular first molar (M1) and its surrounding area were observed at postnatal day 5 (P5), shortly after the time that root morphogenesis of mandibular murine molars had been initiated^17^. At P5, a great majority of dental mesenchymal cells in the dental follicle and papilla were red (Fig. 2a, white arrows: dental follicle, asterisks: dental papilla, sharps: alveolar bones), whereas dental epithelial cells such as ameloblasts and the epithelial root sheath were not labelled (arrowheads), indicating a dental mesenchyme-specific activity of *Osx-cre*. Some distinct non-red mesenchymal populations were also observed, such as in the outer dental follicle between the apex and the alveolar bone (Fig. 2a, yellow arrows), around the furcation area (blue arrow) and in the dental papilla (green arrow). In higher magnifications, dental mesenchymal cells surround the nascent epithelial root sheath (Fig. 2a, lower panel), indicating that Osx-expressing cells and their descendants actively participate in root formation.

To understand how osterix-expressing progenitors at a specific time point contribute to further root morphogenesis, a pulse-chase experiment was conducted using a tamoxifen-inducible *Osx-creER* and an *R26R*-tomato reporter system. *Osx-creER*; *R26R*-tomato mice received tamoxifen at P3 when root morphogenesis was initiated. In this paradigm, only cells actively expressing Osx at P3 undergo recombination in the presence of tamoxifen and become permanently red. Forty-eight hours after a single tamoxifen injection, osterix-expressing red cells were predominantly found among odontoblasts, alveolar bone osteoblasts/cytes and sparsely among dental papilla and follicle cells (Fig. 2b). In higher magnifications, cells in the dental papilla and follicle surrounding the epithelial root sheath were red (Fig. 2b, lower panel, white arrows). We thereafter chased the descendants of these osterix-expressing cells (Osx-P3 cells) for various days to see how these cells contribute to further dental root formation. After 4 days of chase at P7, Osx-P3 cells contributed to an increasing number of dental papilla and follicle cells surrounding the epithelial root sheath (Fig. 2d, arrows), as well as cells in the incipient interradicular area between the roots (arrowhead). Some of Osx-P3 cells were proliferating both in the dental papilla and follicle around the HERS, as they incorporated 5-ethynyl-2′-deoxyuridine (EdU) administered shortly before analysis (Fig. 2d, right panel, yellow arrowheads). In addition, Osx-P3 cells became odontoblasts of the elongating portion of the root structure (Fig. 2d, right panel, asterisks: odontoblasts). After 11 days of chase at P14, Osx-P3 cells participated in root formation by differentiating into a majority of the odontoblasts, dental pulp cells, cementoblasts on the acellular cementum (essential for establishing periodontal attachment) and some PDL cells, especially on the interalveolar septum side (Fig. 2e, arrowheads: cementoblasts, asterisks: odontoblasts, arrows: PDL cells). This trend continued when chased until P25, and Osx-P3 cells substantially contributed to most of aforementioned cell types and cementoblasts on the cellular cementum and their adjacent PDL cells (Fig. 2f, arrowheads: *osteocalcin* (*Oc)*-GFP^+^ (green fluorescent protein) cementoblasts, arrows: PDL cells). *Osx-creER*; *R26R*-tomato mice without tamoxifen injection did not show any accumulation of red cells (Supplementary Fig. 1a–c), suggesting that tamoxifen-independent activity of *Osx-creER* was negligible. Therefore, these data from the lineage-tracing experiments suggest that osterix-expressing cells at P3 include dental mesenchymal progenitors that contribute to all cell types involved in further dental root development.

Type I collagen (Col1) is a most abundant matrix protein in mineralizing tissues produced by differentiated cells, such as osteoblasts, cementoblasts and odontoblasts. To test if Col1-expressing cells contribute to dental root morphogenesis, we undertook a pulse-chase experiment using a tamoxifen-inducible *Col1(3.2 kb)-creER* and an *R26R*-tomato reporter system. Forty-eight hours after injection, Col1-expressing red cells were predominantly found among odontoblasts of the dental crown and alveolar osteoblasts/cytes, but not among dental papilla and follicle mesenchymal cells (Fig. 2c). We sought to chase the descendants of these Col1-expressing cells at P3 (Col1-P3 cells). Unfortunately, we observed substantial tamoxifen-independent activities after P14, especially among odontoblasts and alveolar bone osteoblasts, but not in PDL cells (Supplementary Fig. 1d–f). Therefore, we could not ascertain whether red odontoblasts and alveolar bone osteoblasts represented the descendants of Col1-P3 cells or ongoing 3.2 kb *Col1a1* promoter activities in these cells. Nonetheless, none of dental papilla and follicle mesenchymal cells surrounding the epithelial root sheath was red at P7 (Fig. 2g), and only odontoblasts and alveolar osteoblasts/cytes, but not cementoblasts or PDL cells, were red at P14 and P25 (Fig. 2h,i, asterisks: odontoblasts). Therefore, Col1-expressing cells did not become cementoblasts or PDL cells during further dental root development.

These findings from lineage-tracing experiments suggest that osterix-expressing progenitors, but not mature matrix-producing cells such as odontoblasts and osteoblasts, differentiate into cementoblasts and their adjacent PDL cells during further dental root development.

### Cells in dental follicle and on root surface express PTHrP

**Figure 3:**
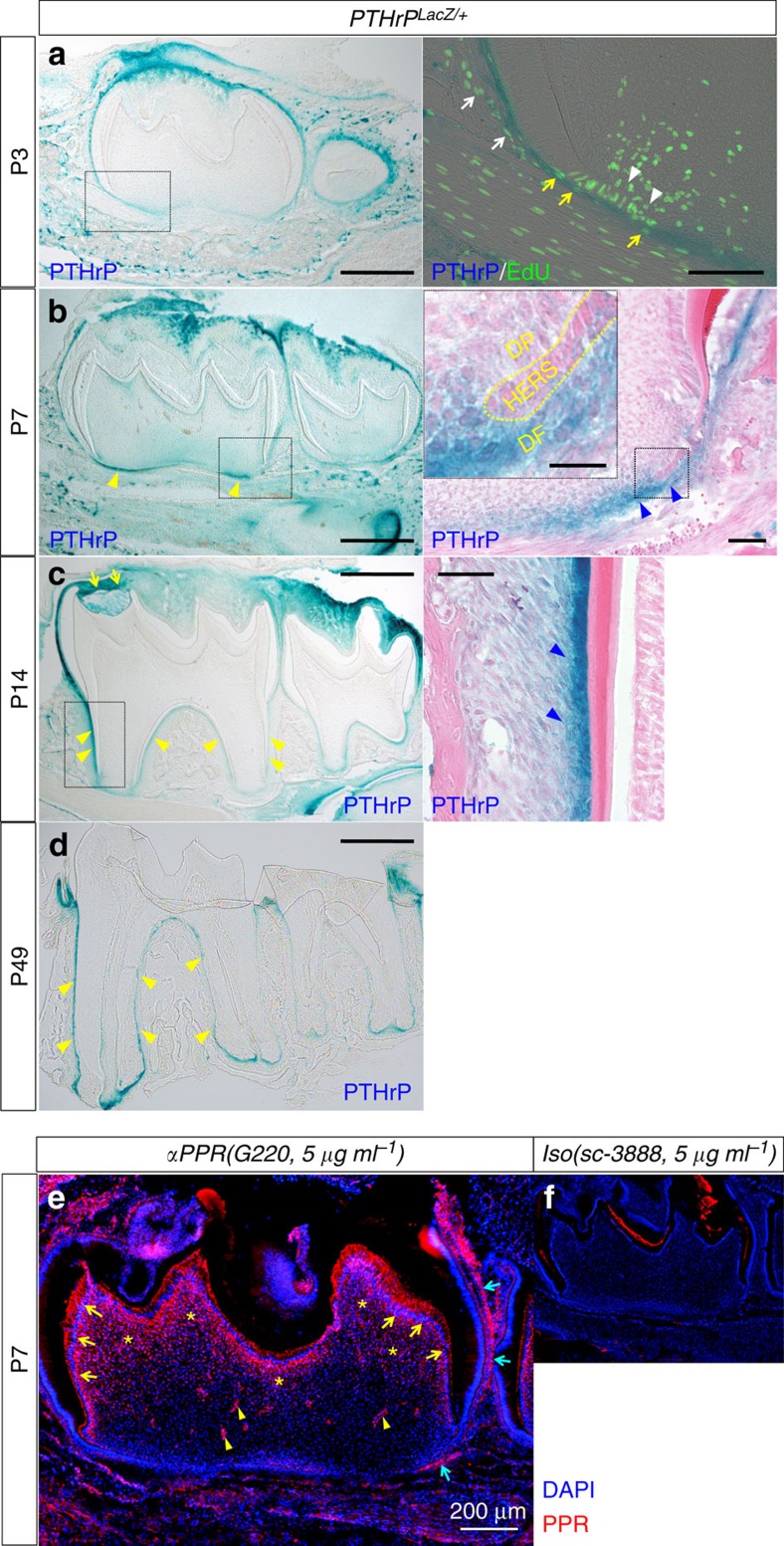
Cells in the dental follicle and the root surface express PTHrP. (**a**) *PTHrP*^*LacZ/+*^ mandibular first molars (M1) sections at P3 were stained for β-galactosidase activity and EdU, which was administered twice (6 and 3 h) prior to analysis. Right panel: a magnified view of the dotted area. Blue: LacZ, green: EdU-Alexa488. White arrowheads: LacZ^−^EdU^+^ dental papilla cells, white arrows: LacZ^−^EdU^+^ dental follicle cells, yellow arrows: LacZ^+^EdU^+^ dental follicle cells. Scale bars: 500 μm (left panel) and 100 μm (right panel). (**b**–**d**) *PTHrP*^*LacZ/+*^ M1 sections were stained for β-galactosidase activity at P7 (**b**), P14 (**c**) and P49 (**d**). Right panels: magnified views of the dotted areas counterstained with eosin. The inset of the right panel shows a further magnified view of the dotted area. Yellow and blue arrowheads: LacZ^+^ dental follicle cells at root formation front, immediately outside HERS (**b**) and on root surface (**c**,**d**). DP: dental papilla, DF: dental follicle. Scale bars: 500 μm (left panels), 50 μm (right panels) and 20 μm (inset). (**e**,**f**) P7 M1 sections were stained for nuclei, anti-PPR antibody (G220) (**e**) or its isotype control (sc-3888) (**f**), both at 5 μg ml^−1^. Yellow arrows: PPR^+^ odontoblasts, yellow arrowheads: PPR^+^ pericytes, yellow asterisks: PPR^+^ dental pulp cells, blue arrows: PPR^+^ dental follicle cells. Red: PPR-Alexa546, blue: DAPI. Scale bar: 200 μm.

To delineate the pattern of PTHrP expression during dental root morphogenesis, we took advantage of a *PTHrP-LacZ* knock-in mouse, in which expression of β-galactosidase is regulated by the endogenous *PTHrP* locus^18^. When root morphogenesis started at P3, PTHrP-expressing blue cells were predominantly found in the dental follicle in a pattern surrounding the tooth (Fig. 2a, blue cells in the surrounding alveolar bone are LacZ-independent activities), but not within the dental papilla and dental pulp. A group of non-blue cells in the dental papilla in proximity to the incipient epithelial root sheath were proliferating as they incorporated EdU administered shortly before analysis (Fig. 3a, arrowheads). In the dental follicle, both blue and non-blue cells were proliferating (Fig. 3a, right panel, white arrows: PTHrP^−^, yellow arrows: PTHrP^+^), indicating that both PTHrP-expressing and non-expressing cells proliferated at the time of the initiation of root formation. When root morphogenesis was well in progress at P7, many dental follicle cells, but many fewer dental papilla cells, were blue at the root formation front (Fig. 3b, yellow arrowheads), suggesting that PTHrP expression during root morphogenesis was rather specific to the portion of the dental follicle that becomes the dental root in the future. In higher magnification, intense *PTHrP-LacZ* activity was found in the dental follicle immediately outside the epithelial root sheath and beyond (Fig. 3b, right panel, blue arrowheads), while its activity was rather weaker in the epithelial root sheath and dental papilla (see inset, Fig. 3b). When the distinct bifurcated roots were formed at P14, PTHrP was still expressed in a pattern surrounding the molar, most evidently in two locations: the dental follicle/sac on the top of the dental crown (Fig. 3c, yellow arrows) and on the root surface (yellow arrowheads). In higher magnification, *PTHrP-LacZ* activities were found in cementoblasts and their adjacent PDL cells (Fig. 3c, right panel, blue arrowheads). When the dental root was completely formed and the first molar completely erupted into the oral cavity at P25 (data not shown) and weeks after that at P49, the root surface-specific pattern of PTHrP expression was maintained (Fig. 3d, yellow arrowheads).

These findings suggest that the mesenchymal cells in the dental follicle and on the root surface robustly express PTHrP during root formation and after tooth eruption, suggesting that this PTHrP may have a role as an important cytokine regulating root morphogenesis and maintenance of periodontal attachment.

### Tooth root formation requires PPR in mesenchymal progenitors

During active root formation, the expression pattern of PTHrP and the distribution of osterix-expressing cells and their descendants (Osx-lineage cells) overlap, particularly in the dental follicle immediately outside the epithelial root sheath (Figs 2a and 3a–d). As the biological range of action for PTHrP is limited, we hypothesize that these Osx-lineage cells in the dental follicle are the principle targets of PTHrP. PPR immunoreactivity was observed broadly among dental mesenchymal cells, such as odontoblasts, coronal dental pulp cells, pericytes and dental follicle cells, but not in dental epithelial cells such as ameloblasts or epithelial root sheath cells, confirming a mesenchyme-specific expression pattern of the PPR in developing molars (Fig. 3e). Unlike in humans, heterozygous inactivation of the PPR in mice did not cause a defect in tooth eruption (Supplementary Fig. 2a,b). To understand the roles of the PPR in dental root-forming mesenchymal cells, we conditionally deleted the PPR using *Osx-cre::*GFP^19^ and the PPR-floxed allele^20^. *PPR*^fl/+, fl/fl^ (Control), *Osx-cre*::GFP; *PPR*^fl/+^ (Osx-PPR cHet) and *Osx-cre*::GFP; *PPR*^fl/fl^ (Osx-PPR conditional knockout (cKO)) littermate mice were analyzed at P3, P7, P18 and P25.

**Figure 4:**
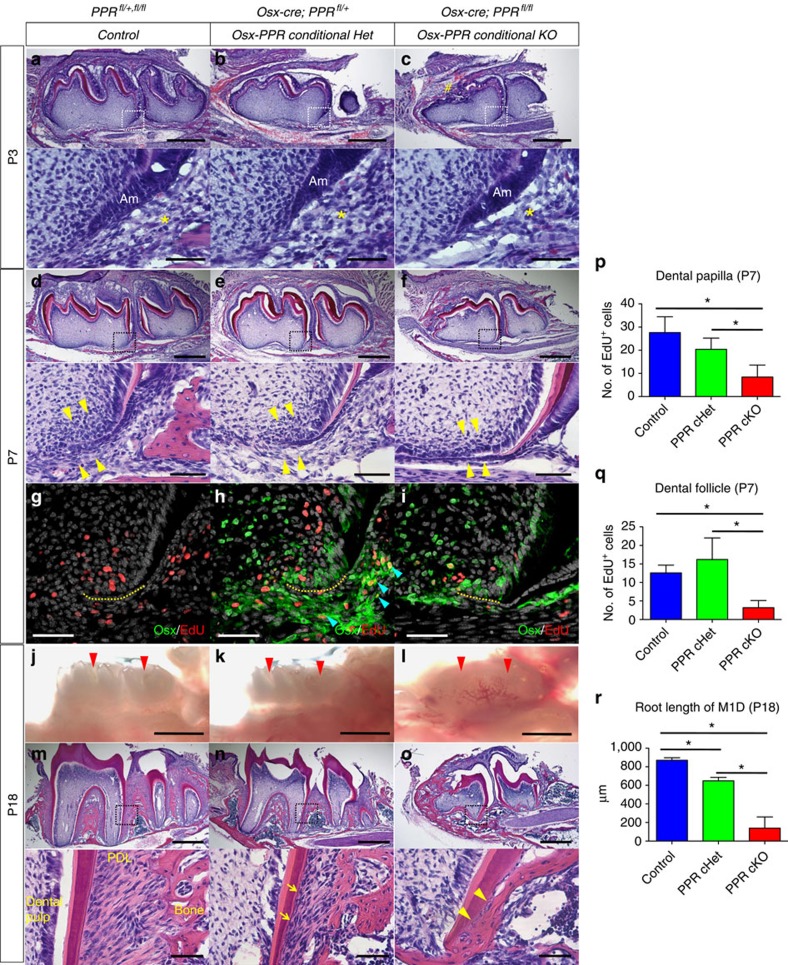
Tooth root formation requires PPR in osterix-lineage cells. (**a**–**f**) Mandibular first molar (M1) sections of (**a**,**d**) Control (*PPR*^fl/+fl/fl^), (**b**,**e**) Osx-PPR conditional Het (*Osx-cre*; *PPR*^fl/+^) and (**c**,**f**) Osx-PPR conditional KO (*Osx-cre*; *PPR*^fl/fl^) mice at P3 (**a**–**c**) and P7 (**d**–**f**) were stained for hematoxylin and eosin. Lower panels: magnified views of the dotted areas. Asterisks: dental follicle, sharp: malformed crown on the mesial portion. Am: ameloblasts, arrowheads: dental papilla and follicle cells around HERS. Scale bars: 500 μm (upper panels) and 50 μm (lower panels). (**g**–**i**) Sections of (**g**) Control, (**h**) Osx-PPR conditional Het and (**i**) Osx-PPR conditional KO molars at P7 were stained for nuclei, EdU and *Osx*-GFP. EdU was administered twice (6 and 3 h) prior to analysis. Green: GFP-Alexa488, red: EdU-Alexa555, grey: DAPI. Yellow dotted lines: HERS, blue arrowheads: GFP^+^EdU^+^ dental follicle cells. Scale bars: 50 μm. (**j**–**l**) Stereoscopic images of (**j**) Control, (**k**) Osx-PPR conditional Het and (**l**) Osx-PPR conditional KO molars at P18 are shown. Red arrowheads: crowns of M1 and M2. Scale bars: 1 mm. (**m**–**o**) M1 sections of (**m**) Control, (**n**) Osx-PPR conditional Het and (**o**) Osx-PPR conditional KO molars at P18 were stained for hematoxylin and eosin. Lower panels: magnified views of the dotted areas. Arrows: irregular cementum, arrowheads: ankylosis of cementum to bone. Scale bars: 500 μm (upper panels) and 50 μm (lower panels). (**p**,**q**) Shown is quantification of EdU^+^ cells in dental papilla (**p**) and dental follicle (**q**) around HERS at P7. Blue bars: Control, green bars: Osx-PPR conditional Het, red bars: Osx-PPR conditional KO, *n*=5 per group, *P<0.05, Mann–Whitney's *U*-test. (**r**) Shown is quantification of M1 distal root length at P18. Blue bars: Control, green bars: Osx-PPR conditional Het, red bars: Osx-PPR conditional KO. *n*=4 per group, *P<0.05, Mann–Whitney's *U*-test All data are represented as mean±s.d.

At P3, the time that root formation was initiated, the dental follicle surrounding apical ameloblasts was comparable among the three genotypes (Fig. 4a–c, lower panels, yellow asterisks), although the mesial portion of the crown was malformed in the Osx-PPR conditional knockout mice (Fig. 4c, sharp). When root formation was in progress at P7, mesenchymal cells of the dental papilla and follicle were densely recruited around the epithelial root sheath in wild-type (WT) control teeth (Fig. 4d, yellow arrowheads). In the Osx-PPR conditional Het, the width of the dental follicle was comparable to control, although dental follicle cells adjacent to the epithelial root sheath appeared to be sparse (Fig. 4e). In the Osx-PPR conditional knockout, the dental follicle was narrower than in controls, and dental follicle cells around the epithelial root sheath were far fewer than in the conditional het (Fig. 4f). Cell proliferation was evaluated by administering EdU to these mice shortly before analysis. In both WT control and the Osx-PPR conditional Het, EdU^+^ dental papilla and follicle mesenchymal cells were observed surrounding the epithelial root sheath (Fig. 4g,h, yellow dotted line: epithelial root sheath). However in the Osx-PPR conditional knockout, the number of EdU^+^ cells was significantly reduced both in the dental papilla and follicle (Fig. 4i, quantification of EdU^+^ dental papilla and follicle cells is shown in Fig. 4p,q, Mann–Whitney's *U*-test), suggesting that complete deletion of the PPR led to impaired proliferation of these mesenchymal cells.

To ascertain whether the defect in proliferation occurs in osterix-expressing progenitors, we conducted anti-GFP staining in addition to EdU detection. Cells actively expressing *Osx-cre*::GFP are detected as green, while the nucleus of proliferating cells become red. The GFP signal was not observed in WT control sections, confirming the specificity of the antibody (Fig. 4g). In the Osx-PPR conditional Het, a number of dental mesenchymal cells surrounding the epithelial root sheath were green, and proliferating Osx^+^ cells were especially observed in the dental follicle (Fig. 4h, blue arrowheads). In the PPR conditional knockout, proliferation of Osx^+^ cells in the dental follicle was abrogated (Fig. 4i).

At P18, mandibular molars of WT control and the Osx-PPR conditional Het erupted normally into the oral cavity (Fig. 4j,k). However, in the Osx-PPR conditional knockout mice, molars did not erupt and were covered by the dental sac (Fig. 4l). In WT control mice, the dental root was normally formed with its length longer than the crown, and the PDL was highly organized with oblique fibres extending into the acellular cementum on the root surface (Fig. 4m). In the Osx-PPR conditional Het, the dental root was slightly shorter than in the control, accompanied with thinner and less organized PDL as well as irregular cementum (Fig. 4n, lower panel, arrows). In the Osx-PPR conditional knockout, the dental root was significantly truncated, making it virtually rootless (Fig. 4o, quantification of the root length of M1D is shown in Fig. 4r, Mann–Whitney's *U*-test). These molars lacked the PDL, with the root cementum appearing to be ankylosed to the surrounding hypomorphic bones (Fig. 4o). Most of the Osx-PPR conditional knockout mice died after weaning for uncertain reasons. *Osx-cre*::GFP; PPR^+/+^ molars did not show an overt phenotype (Supplementary Fig. 2c,d), indicating that the presence of *Osx-cre*::GFP was not responsible for the root truncation. We further performed tartrate-resistant acid phosphatase (TRAP) staining to ascertain if alterations in osteoclasts accounted for the phenotype. No apparent difference was observed in terms of the location and the number of TRAP^+^ cells around molars before and after root formation and eruption (Supplementary Fig. 2e–m), suggesting that defect in osteoclasts was not the principal cause of these phenotypes.

**Figure 5:**
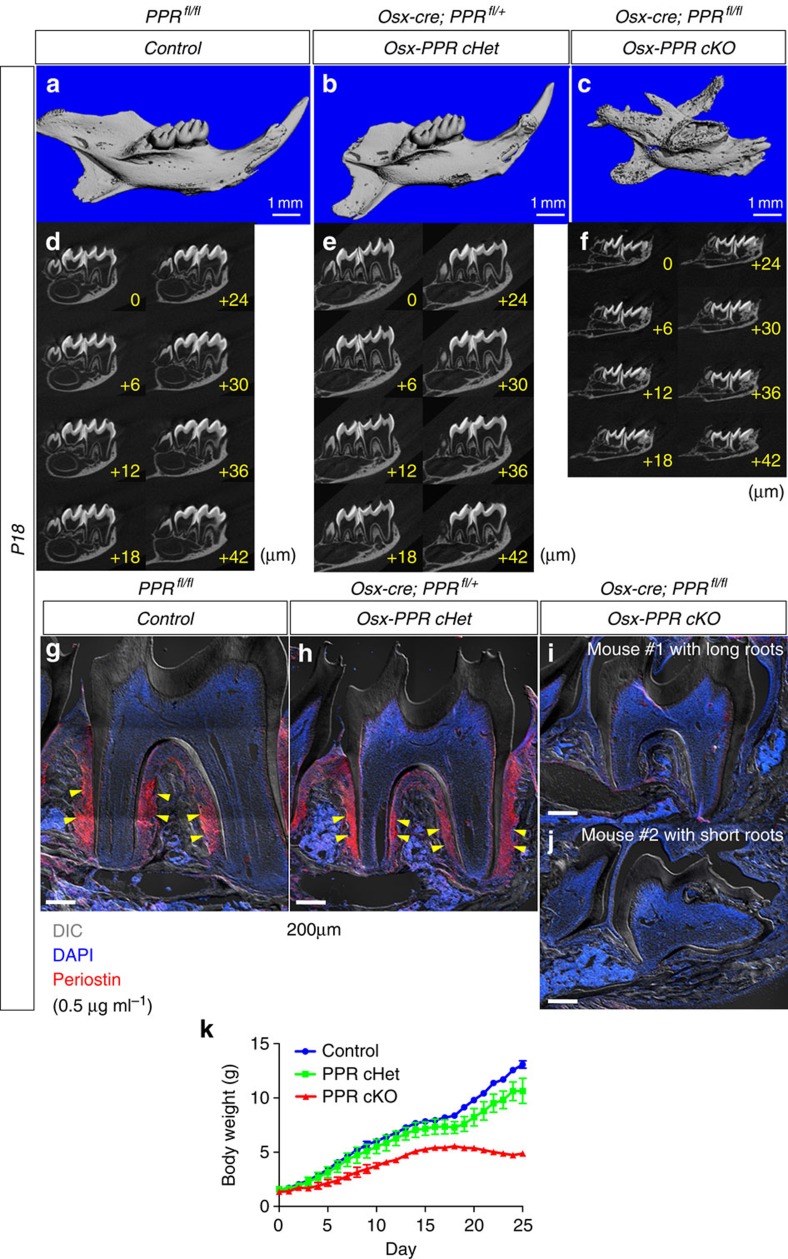
Tooth eruption and periostin expression requires PPR in osterix-lineage cells. (**a**–**f**) 3D microCT images of (**a**,**d**) Control, (**b**,**e**) Osx-PPR conditional Het and (**c**,**f**) Osx-PPR conditional KO molars at P18 are shown. Upper panels: 3D reconstructed microCT images of the mandible, lower panels: individual slices of microCT images at an interval of 6 μm. Scale bars: 1 mm. (**g**,**h**) Sections of (**g**) Control, (**h**) Osx-PPR conditional Het and (**i**,**j**) Osx-PPR conditional KO molars at P18 were stained for nuclei and anti-periostin (ABT280) at 0.5 μg ml^−1^. Osx-PPR conditional KO mice with long roots (Mouse #1) (**i**) and short roots (Mouse #2) (**j**) are shown. Red: Periostin-Alexa546, blue: DAPI, grey: DIC (differential interference contrast). Yellow arrowheads: periostin^+^ PDL cells in the middle portion of the dental root. Scale bars: 200 μm. (**k**) Body weight measurement of Control (blue line), Osx-PPR conditional Het (green line) and Osx-PPR conditional KO (red line) mice between P0 and P25. *n*=4–16 per group, data are represented as mean±s.d.

Subsequently, we performed a 3D microcomputed tomography (microCT) imaging analysis of littermates with three corresponding genotypes at P18. Consistent with macroscopic findings, mandibular molars of the WT Control and the Osx-PPR conditional Het erupted into the oral cavity (Fig. 5a,b), whereas those of the Osx-PPR conditional KO did not erupt and were covered by the cortical shell (Fig. 5c). Individual slices of microCT images at an interval of 6 μm revealed that the dental root was normally formed in the WT Control and the Osx-PPR cHet (Fig. 5d,e), whereas it was truncated in the Osx-PPR cKO (Fig. 5f). These data confirm the reproducibility of our 2D histological analyses in 3D microCT imaging analyses.

We also performed immunohistochemical staining for periostin as a marker for PDLs at P18. As previously reported^21^, strong immunoreactivity for periostin was particularly observed in the PDL, most notably in the middle portion of the dental root among periodontal fibroblasts and cementoblasts, (Fig. 5g,h, yellow arrowheads). No immunoreactivity was noted within the dental pulp or alveolar bones. In contrast, in the Osx-PPR conditional KO, no periostin immunoreactivity was observed in areas surrounding the dental roots, regardless of the length of the roots (Fig. 5i,j, see #1 long vs #2 short roots). These findings indicate that PPR signalling in Osx-expressing dental mesenchymal progenitors is essential for their differentiation into periostin-expressing PDL cells.

We also measured the body weight of three corresponding genotypes beginning the time of birth (P0) until P25 (Fig. 5k). The Osx-PPR cKO mice consistently showed lower body weight than the WT Control and the Osx-PPR cHet mice throughout postnatal growth. In addition, the Osx-PPR cHet consistently showed slightly lower body weight than the WT Control. After weaning at P21, the discrepancy of the body weight among three genotypes was enhanced, probably due to the tooth phenotype.

These data demonstrate that deletion of the PPR in Osx-lineage cells causes a defect in proliferation of dental mesenchymal cells around the epithelial root sheath, in association with agenesis of the PDL, ankylosis of the dental root to the alveolar bone and truncation of the dental root. Although underlying alveolar bones of the Osx-PPR cKO mice were underdeveloped compared to those in the other genotypes, we think this represents an independent consequence of the genetic mutation. However, as dental root formation is closely coupled with alveolar bone formation, these two biological events cannot be effectively segregated. Therefore, we cannot eliminate the possibility that abnormalities of the dental root and the mandible influence each other.

### Tooth root formation does not require PPR in mature cells

**Figure 6:**
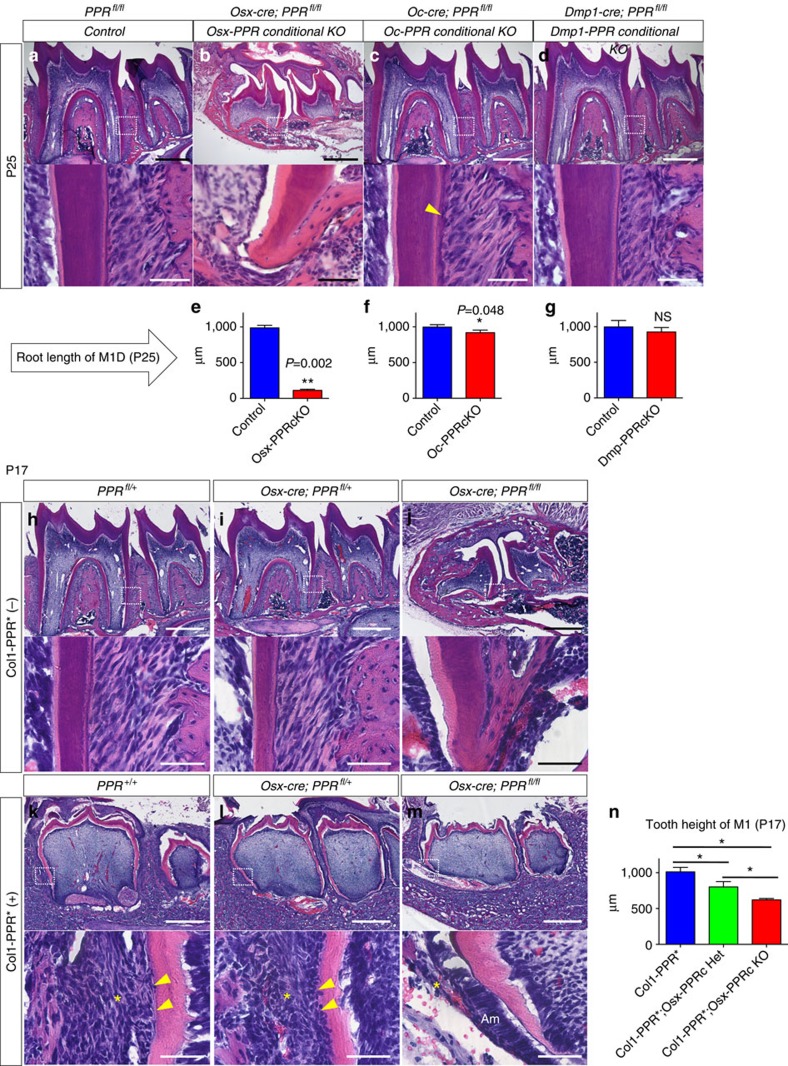
PPR in differentiated matrix-producing cells is dispensable for tooth root formation. (**a**–**d**) Mandibular first molar (M1) sections of (**a**) Control (*PPR*^fl/fl^), (**b**) Osx-PPR conditional KO (*Osx-cre*; *PPR*^fl/fl^), (**c**) Oc-PPR conditional KO (*Oc-cre*; *PPR*^fl/fl^) and (**d**) Dmp1-PPR conditional KO (*Dmp1-cre*; *PPR*^fl/fl^) mice at P25 were stained for hematoxylin and eosin. Lower panels: magnified views of the dotted areas. Yellow arrowhead: embedded cementocyte. Scale bars: 500 μm (upper panels) and 50 μm (lower panels). (**e**–**g**) Shown is quantification of M1 distal root length at P25. Blue bars: Control, red bars: (**e**) Osx-PPR conditional KO, (**f**) Oc-PPR conditional KO and (**g**) Dmp1-PPR conditional KO mice. Control: *n*=3–6 per group, conditional KO: *n*=6–7 per group, *P<0.05, **P<0.01, Mann–Whitney's *U*-test. (**h**–**m**) M1 sections of (**h**) *PPR*^*fl/+*^, (**i**) *Osx-cre; PPR*^*fl/+*^, (**j**) *Osx-cre; PPR*^*fl/fl*^, (**k**) Col1-PPR*; *PPR*^*+/+*^, (**l**) Col1-PPR*; *Osx-cre*; *PPR*^*fl/+*^ and (**m**) Col1-PPR*; *Osx-cre*; *PPR*^*fl/fl*^ molars at P17 were stained for hematoxylin and eosin. Lower panels: magnified views of the dotted areas. Arrowheads: cementoblasts, asterisks: PDL/dental follicle. Scale bars: 500 μm (upper panels) and 50 μm (lower panels). (**n**) Shown is quantification of M1 tooth height at P17. Blue bars: Col1-PPR* Control, green bars: Col1-PPR*; Osx-PPR conditional Het, red bars: Col1-PPR*; Osx-PPR conditional KO. *n*=4–5 per group, *P<0.05, Mann–Whitney's *U*-test. All data are represented as mean±s.d.

The Osx-PPR conditional knockout mice showed significantly shorter dental roots than WT controls at P25 (Fig. 6b, quantification of the root length of M1D is shown in Fig. 6e, Mann–Whitney's *U*-test). To determine the roles of the PPR specifically in differentiated mineralizing cells during dental root formation, we conditionally deleted the PPR using *osteocalcin (Oc)-cre*^22^ and *Dmp1-cre*^23^. *PPR*^fl/fl^ (Control) and *Oc-cre*; *PPR*^fl/fl^ (Oc-PPR cKO) or *Dmp1-cre*; *PPR*^fl/fl^ (Dmp-PPR cKO) littermate mice were analyzed at P25.

Interestingly, fate mapping using an *R26R*-tomato revealed that *Oc-cre* and *Dmp1-cre* marked different mineralizing cell types in molar roots. While both *cre* lines similarly targeted a majority of alveolar osteoblasts/cytes, *Oc-cre* targeted more cementoblasts (arrowheads, Supplementary Fig. 3d,e) than odontoblasts (asterisks, Supplementary Fig. 3d,e), whereas *Dmp1-cre* targeted more odontoblasts than cementoblasts. Unlike *Osx-cre*, both *Oc-cre* and *Dmp1-cre* did not target dental pulp cells and PDL cells (Supplementary Fig. 3b,c). The Oc-PPR conditional knockout mice exhibited slightly shorter roots than controls (Fig. 6c,f). The cementum of the Oc-PPR conditional knockout appeared to be irregular and thicker, with occasional embedded cementocytes in the portion of the root not observed in WT controls (Fig. 6c, lower panel, arrowhead). Dmp1-PPR conditional knockout mice did not show any significant alteration of cell proliferation around the HERS (Supplementary Fig. 3h–k, Mann–Whitney's *U*-test) or any significant root truncation, and showed no overt alteration in the cementum (Fig. 6d,g, Mann–Whitney's *U*-test). Therefore, these data revealed that deletion of the PPR in differentiated matrix-producing cells leads to only minor to moderate truncation of dental roots and abnormal cementum, suggesting that the PPR in matrix-producing cells is not essential for dental root formation and may have effects directly in cementoblasts that affect cementum.

To investigate further whether an activated PPR expressed specifically in differentiated matrix-producing cells can rescue the root truncation phenotype of the Osx-PPR conditional knockout mice, we took advantage of *Col1a1-caPPR* transgenic mice^24^ in which a constitutively activated PPR with the Jansen-type constitutively active mutation is expressed in Col1^+^ matrix-producing cells including odontoblasts, cementoblasts and alveolar bone osteoblasts (Supplementary Fig. 3f,g). Littermates with six genotypes, *PPR*^+/+^, *PPR*^fl/+,fl/fl^ (Control), *Osx-cre*; *PPR*^fl/+^ (Osx-PPR cHet), *Osx-cre*; *PPR*^fl/fl^ (Osx-PPR cKO), *PPR*^+/+,fl/+,fl/fl^; *Col1a1-caPPR* (Col1-PPR*), *Osx-cre; PPR*^fl/+^; *Col1a1-caPPR* (Osx-PPR cHet; Col1-PPR*) and *Osx-cre; PPR*^fl/fl^; *Col1a1-caPPR* (Osx-PPR cKO; Col1-PPR*) were analyzed at P17.

Mandibular molars of the Col1-PPR* exhibited a widened dental pulp associated with thin and undermineralized enamel and dentin^25^, and did not erupt into the oral cavity at P17 (Fig. 6k). The roots of these molars were not bifurcated, and were morphologically different from WT controls. Nonetheless, an immature PDL and cementum were formed on the root surface of these transgenic molars (Fig. 6k, lower panel, arrowheads: cementoblasts, asterisks: PDL). In the Osx-PPR cHet; Col1-PPR*, although the height of mandibular molars was shorter than those in the Col1-PPR*, an immature PDL and cementum were still formed on the root surface, particularly on the mesial side (Fig. 6l, lower panel). In the Osx-PPR cKO; Col1-PPR*, the height of mandibular molars was even shorter than the Osx-PPR cHet; Col1-PPR*, and the root was completely absent without any PDL or cementum (Fig. 6m), while the crown morphology was comparable to that of the Col1-PPR*. The outer dentin surface of the Osx-PPR cKO; Col1-PPR* molars was covered by the dental epithelium consists of ameloblastic cells, and its adjacent dental follicle was underdeveloped (Fig. 6m, asterisk: dental follicle, quantification of the tooth height of M1 is shown in Fig. 6n).

These findings suggest that constitutive activation of the PPR in differentiated matrix-producing cells rescues the crown phenotype, but not the root phenotype of the Osx-PPR conditional knockout mice. In fact, constitutive activation of the PPR in differentiated matrix-producing cells appears to interfere with the function of root-generating cells. These observations support the idea that the PPR signalling in Osx-expressing progenitors before their differentiation into Col1-expressing cells is essential for initiation of dental root formation.

### PPR orchestrates cementoblast differentiation of progenitors

To further understand how the defective PPR signalling affects osterix-expressing root-forming progenitors in a cell-autonomous manner, we conducted a lineage-tracing experiment of the PPR-deficient cells using a tamoxifen-inducible *Osx-creER*, an *R26R*-tomato reporter locus and the PPR-floxed alleles. The triple mutant mice (*Osx-creER*; *R26R*-tomato; *PPR*^fl/fl^) and their creER-negative control mice received tamoxifen at P3 when root morphogenesis was initiated. In this system, only cells actively expressing Osx at P3 undergo recombination in the presence of tamoxifen; in these cells, *cre* recombinase removes the “stop” sequences in the Rosa26 locus (allowing tomato expression) and the floxed exon E1 in the *PPR* locus (eliminating PPR function). As a result, osterix-expressing cells at P3 and their descendants become red and deficient for the receptor (PPR temporal conditional KO (Osx-PPR tcKO)), making it possible to trace the fate of PPR-deficient cells as the root develops. In addition, we also conducted a similar experiment using the triple mutant mice (*Col1-creER*; *R26R*-tomato; *PPR*^fl/fl^), in which Col1-expressing cells at P3 and their descendants become red and deficient for the receptor (Col1-PPR tcKO).

**Figure 7:**
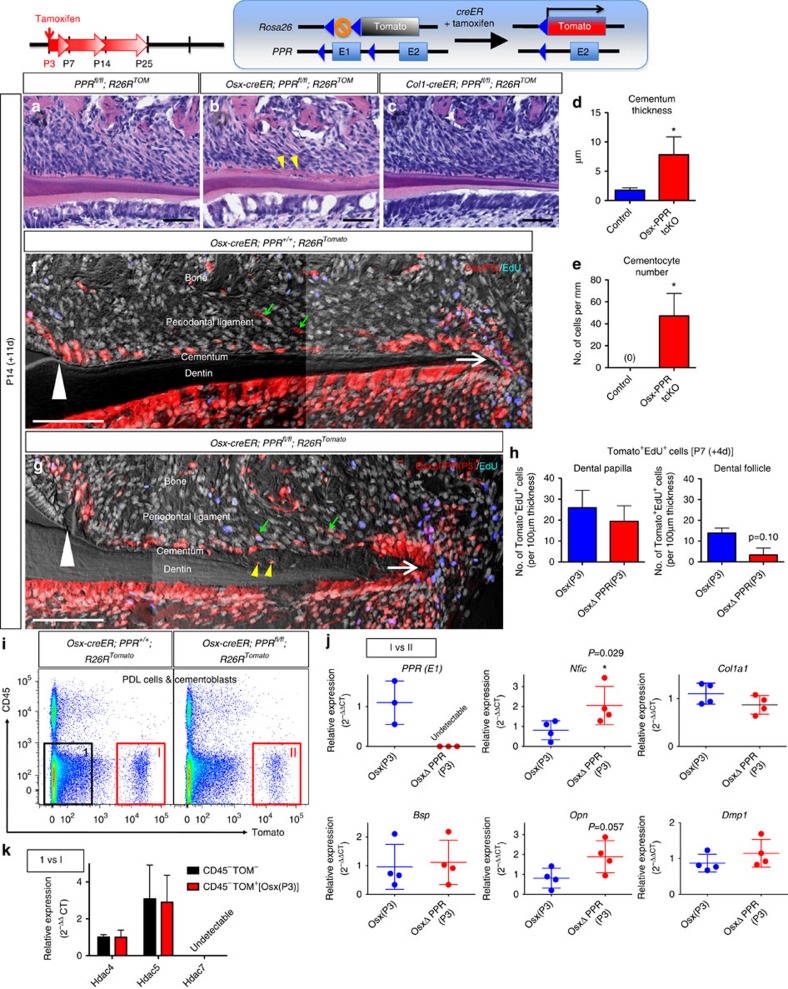
PPR orchestrates cementoblast differentiation of osterix-expressing progenitors. (**a**–**c**) *PPR*^fl/fl^; *R26R*^Tomato^ (**a**), *Osx-creER*; *PPR*^fl/fl^; *R26R*^Tomato^ (**b**) and *Col1-creER*; *PPR*^fl/fl^; *R26R*^Tomato^ (**c**) mice received tamoxifen at P3 and were analyzed at P14. Mandibular first molar (M1) sections were stained for hematoxylin and eosin, and crown is on the left. Yellow arrowheads: cellular cementum. Scale bars: 50 μm. (**d**,**e**) Shown is quantification of M1 distal root cementum thickness (**d**) and cementocyte number (**e**) at P14 (tamoxifen at P3). Blue bars: Control (*PPR*^fl/fl^; *R26R*^Tomato^), red bars: Osx-PPR tcKO (*Osx-creER*; *PPR*^fl/fl^; *R26R*^Tomato^). *n*=4 per group, *P<0.05, Mann–Whitney's *U*-test. (**f**,**g**) *Osx-creER*; *PPR*^+/+^; *R26R*^Tomato^ (**f**) and *Osx-creER*; *PPR*^fl/fl^; *R26R*^Tomato^ (**g**) mice received tamoxifen at P3 and were analyzed at P14. EdU was administered twice (6 and 3 h) prior to analysis. M1 sections were stained for nuclei and EdU. Blue: EdU-Alexa647, red: tdTomato, grey: DAPI and DIC. White arrowheads: cementoenamel junction, white arrows: apex, green arrows: Tomato^+^ PDL cells, yellow arrowheads: Tomato^+^ embedded cementocytes. Scale bars: 100 μm. (**h**) Shown is quantification of Tomato^+^EdU^+^ cells in dental papilla (left panel) and dental follicle (right panel) around HERS at P7 (tamoxifen was injected at P3). Blue bars: EdU^+^Osx(P3) (*Osx-creER*; *PPR*^+/+^; *R26R*^Tomato^) cells, red bars: EdU^+^OsxΔPPR(P3) (*Osx-creER*; *PPR*^fl/fl^; *R26R*^Tomato^) cells. Double positive cells were counted on 10–15 serial confocal sections of each molar. *n*=3 per group. (**i**–**k**) *Osx-creER*; *PPR*^+/+^; *R26R*^Tomato^ (**f**) and *Osx-creER*; *PPR*^fl/fl^; *R26R*^Tomato^ (**g**) mice received tamoxifen at P3 and molars (M1, M2) were extracted at P25. Collagenase-digested PDL cells and cementoblasts were stained for CD45 and subjected to FACS analysis. (**i**) A representative FACS plot of FSC/SSC-gated singlets. Left panel: cells from *Osx-creER*; *PPR*^+/+^; *R26R*^Tomato^, right panel: cells from *Osx-creER*; *PPR*^fl/fl^; *R26R*^Tomato^. *X* axis: tdTomato, *Y* axis: CD45. Fraction [1]: CD45^−^Tomato^−^ *PPR*^+/+^, [I]: CD45^−^Tomato^+^*PPR*^+/+^ and [II]: CD45^−^Tomato^+^Δ*PPR*^fl/fl^ was sorted to isolate RNA. (**j**) mRNA expression levels of *PPR (E1)*, *Nfic*, *Col1a1*, *Bsp*, *Opn* and *Dmp1* in fraction [I] and [II] determined by qPCR. (**k**) mRNA expression levels of *Hdac4*, *Hdac5* and *Hdac7* in fraction [1] and [I] determined by qPCR. All data were normalized to β*-actin* (*Actb*). *n*=3–4 per group, *P<0.05, Mann–Whitney's *U*-test. All data represented as mean±s.d.

After 11 days of chase at P14 when the root was half-formed, the cementum of WT controls and the Col1-PPR tcKO was thin, acellular and organized on the root surface (Fig. 7a,c, see also Supplementary Fig. 4c,d). In contrast, the cementum was significantly thicker in the Osx-PPR tcKO, with a number of cementocytes embedded within the matrix (Fig. 7b, quantification of the cementum thickness and cementocyte number are shown in Fig. 7d,e, Mann–Whitney's *U*-test). No overt phenotype was observed in the dentin and the dental pulp of the Osx-PPR tcKO, although odontoblasts appeared to be sparse on the dentin surface (Fig. 7b). Dark field images revealed that the Osx-P3 WT red cells contributed to cementoblasts on the root surface all the way from the cementoenamel junction (Fig. 7f, white arrowheads) to the apex (white arrows), and to some adjacent PDL cells (green arrows). The Osx-P3 PPR-deficient red cells also became cementoblasts along with the entire root surface and, occasionally, embedded in the cementum matrix (Fig. 7g, yellow arrowheads). Proliferation of the Osx-P3 PPR-deficient red cells was comparable to that of Osx-P3 PPR WT cells in the dental papilla, but exhibited a trend toward a decrease of proliferation in the dental follicle around the HERS after 4 days of chase at P7 (Fig. 7h, see also Supplementary Fig. 4a,b for images), suggesting that PPR-deficiency moderately affects proliferation of dental follicle cells in a cell-autonomous manner. Osx-PPR tcKO and Col1-PPR tcKO molars erupted normally into the oral cavity, and the root length of their mandibular first molar of the Osx-PPR tcKO appeared to be slightly shorter than that of controls (Supplementary Fig. 4e–h).

To further identify genes responsible for this cementum phenotype caused by the PPR-deficient cementoblast progenitors, we performed fluorescence-activated cell sorting (FACS) of PDL cells and cementoblasts harvested from Osx-P3 WT (*Osx-creER*; *R26R*-tomato) or Osx-P3 PPR tcKO (*Osx-creER*; *R26R*-tomato; *PPR*^fl/fl^) molars. After 22 days of chase at P25, maxillary and mandibular first and second molars were extracted and collagenase-digested, and the released cells were analyzed on the flow cytometer. We confirmed that only PDL cells and cementoblasts, but not dental pulp cells, odontoblasts or osteoblasts, were harvested by this protocol^26^ (Supplementary Fig. 5). Non-hematopoietic CD45^−^ cells consists of 89.5±5.2% (Osx-P3 WT) and 83.7±4.6% (Osx-P3 PPR tcKO) of harvested single cells, and within the CD45^−^ fraction, 2.1±0.7% and 2.0±0.5% were Tomato^+^ in Osx-P3 WT and Osx-P3 PPR tcKO molars, respectively (Fig. 7i). We further sorted Tomato^+^ cells and isolated RNA to analyze gene expression profiles using quantitative PCR (qPCR) (each point in the figure represents an independent biological sample). Importantly, *PPR* messenger RNA (mRNA) was undetectable in Osx-P3 PPR^fl/fl^ Tomato^+^ cells (Fig. 7j), suggesting that these Tomato^+^ cells lack *PPR* at least on an mRNA level. In the Osx-P3 PPR-deficient Tomato^+^ cells, mRNA expression of the bone/cementum matrix protein, osteopontin (*Opn*), showed a trend toward upregulation compared to Osx-P3 PPR WT cells, although this result was not statistically significant (Fig. 7j, Mann–Whitney's *U*-test). In addition, we found that nuclear factor I/C *Nfic*, whose deficiency leads to truncation of molar roots^27^, was upregulated in the Osx-P3 PPR-deficient cells (Fig. 7j).

These data suggest that Osx-expressing root-forming progenitors lacking the PPR can still proliferate and differentiate into odontoblasts, cementoblasts and PDL cells during root formation. However, the PPR-deficient progenitors exhibited accelerated cementoblast differentiation with upregulation of *Nfic*, leading to the formation of cellular cementum on the root surface where acellular cementum is normally formed.

### HDAC4 mutants recapitulate PPR mutant root phenotypes

**Figure 8:**
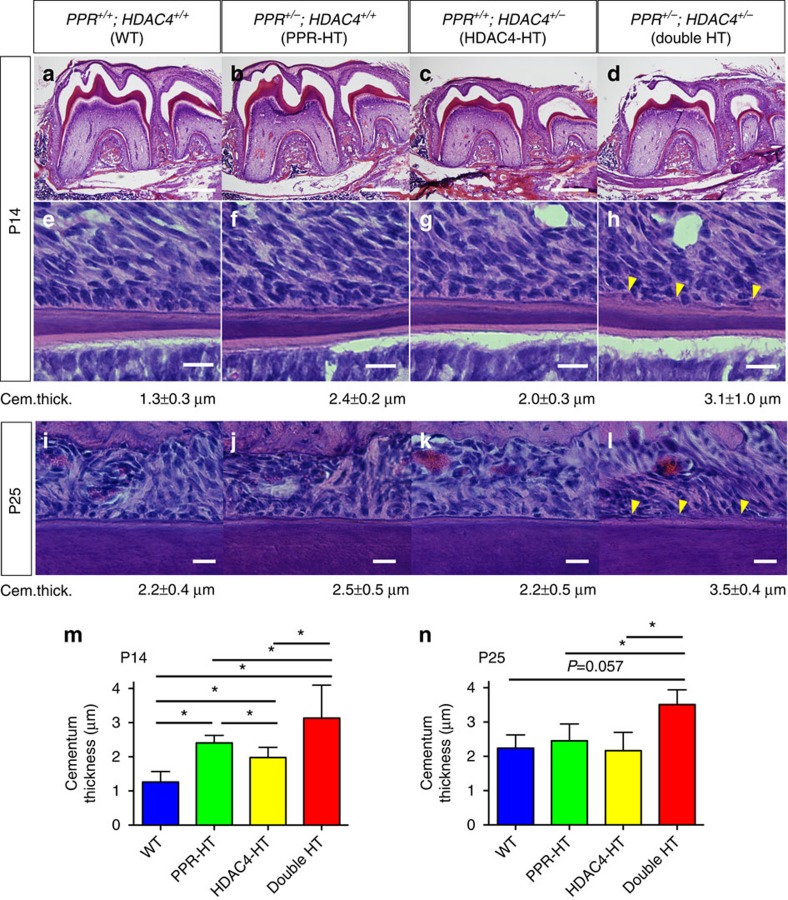
Genetic interactions of PPR and HDAC4 in tooth root phenotypes. (**a**–**h**) Mandibular first molar (M1) sections of (**a**,**e**) WT (*PPR*^+/+^; *HDAC4*^+/+^), (**b**,**f**) PPR-HT (*PPR*^+/−^; *HDAC4*^+/+^), (**c**,**g**) HDAC4-HT (*PPR*^+/+^; *HDAC4*^+/−^) and (**d**,**h**) double HT (*PPR*^+/−^; *HDAC4*^+/−^) mice at P14 were stained for hematoxylin and eosin. Lower panels: magnified views of the middle portion of the M1 distal roots. Yellow arrowheads: rough/disorganized cementum. The cementum thickness of each group is shown below the lower panels. Scale bars: 500 μm (upper panels), 20 μm (lower panels). (**i**–**l**) M1 sections of (**i**) WT, (**j**) PPR-HT, (**k**) HDAC4-HT and (**l**) double HT mice at P25 were stained for hematoxylin and eosin. Shown are magnified views of the middle portion of the M1 distal roots. Yellow arrowheads: rough/disorganized cementum. The cementum thickness of each group is shown below the lower panels. Scale bars: 20 μm. (**m**,**n**) Shown are quantification of M1 distal root cementum thickness at P14 (**m**) and P25 (**n**). *n*=3–9 per group, *P<0.05, Mann–Whitney's *U*-test. All data represented as mean±s.d.

HDAC4 has been proposed to be a candidate downstream mediator of the PPR–Gsα–cAMP–protein kinase A pathway^11^,^28^,^29^. On the basis of our qPCR analysis on sorted PDL cells and cementoblasts, we found that, among Class II HDACs, *Hdac4* and *Hdac5*, but not *Hdac7*, were expressed in these cells (Fig. 7k). To understand possible involvement of HDAC4 in the PPR signalling in dental mesenchymal progenitors and root formation, we first performed genetic experiments by crossing PPR^+/−^ and HDAC4^+/−^ mice. Littermates with four corresponding genotypes, PPR^+/+^; HDAC4^+/+^ (WT), PPR^+/−^; HDAC4^+/+^ (PPR-heterozygote (HT)), PPR^+/+^; HDAC4^+/−^ (HDAC4-HT) and PPR^+/−^; HDAC4^+/−^ (DHT) were analyzed at P14 and P25. At P14, no significance difference was noted in the root length among the four genotypes (Fig. 8a–d). There was an additive effect of double heterozygosity on the cementum thickness at P14 (Fig. 8m). The cementum of the WT control was thin (1.3±0.3 μm) and smooth on the root surface (Fig. 8e). Both the PPR-HT and HDAC4-HT mice exhibited an increased cementum thickness compared to the WT control (2.4±0.2 μm and 2.0±0.3 μm, respectively), with the former genotype showing an increased thickness compared to the latter (Fig. 8f,g). Despite an increased thickness, no apparent alteration in the structure or organization of the cementum was noted in these single heterozygous mice. In the double HT mice, the cementum thickness was further increased compared to the single heterozygous mice or the WT control (3.1±1.0 μm, Fig. 8h). More strikingly, unlike the other three genotypes, the double HT mice exhibited a rough and disorganized cementum with cementocytes occasionally embedded in the matrix (Fig. 8h, arrowheads). This phenotype is reminiscent of the Osx-PPR tcKO cementum at P14 (Fig. 7b). At P25, there was a synergistic effect of double heterozygosity on the cementum thickness (Fig. 8n). In the double HT mice, the cementum thickness was increased compared to the single heterozygous mice, and exhibited a rough and disorganized cementum unlike the other three genotypes, as seen at P14 (Fig. 8i–l). No obvious embedded cementocyte was noted in the double HT mice that we examined. Therefore, the qualitative difference (roughness/smoothness at P14 and P25) and the thickness (at P25) of the cementum may suggest functional interactions of PPR and HDAC4 in dental root formation.

**Figure 9:**
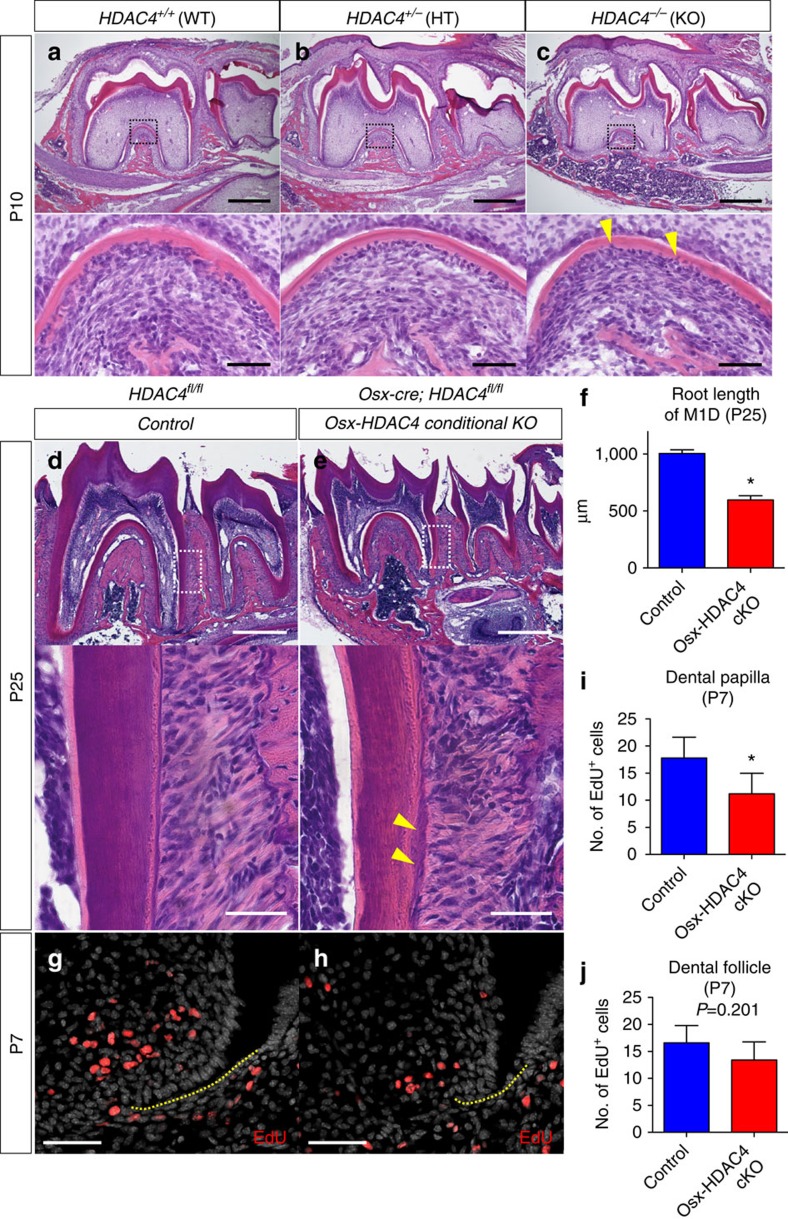
Deletion of HDAC4 partially recapitulates the PPR-deficient root phenotype. (**a**–**c**) Mandibular first molar (M1) sections of (**a**) *HDAC4*^+/+^ (WT), (**b**) *HDAC4*^+/−^ (HT) and (**b**) *HDAC4*^−/−^ (KO) at P10 were stained for hematoxylin and eosin. Lower panels: magnified views of dotted areas. Yellow arrowheads: embedded cementocytes. Scale bars: 500 μm (upper panels) and 50 μm (lower panels). (**d**,**e**) Mandibular first molar (M1) sections of (**a**) Control (*HDAC4*^fl/fl^) and (**b**) Osx-HDAC4 conditional KO (*Osx-cre*; *HDAC4*^fl/fl^) mice at P25 were stained for hematoxylin and eosin. Lower panels: magnified views of the dotted areas. Yellow arrowheads: thick and irregular cementum. Scale bars: 500 μm (upper panels) and 50 μm (lower panels). (**f**) Shown is quantification of M1 distal root length at P25. Blue bars: Control, red bars: Osx-HDAC4 conditional KO. *n*=4 per group. (**g**,**h**) Sections of (**g**) Control (*HDAC4*^fl/fl^) and (**h**) Osx-HDAC4 conditional KO (*Osx-cre*; *HDAC4*^fl/fl^) mice at P7 were stained for nuclei and EdU. EdU was administered twice (6 and 3 h) prior to analysis. Red: EdU-Alexa555, grey: DAPI. Yellow dotted lines: HERS. Scale bars: 50 μm. (**i**,**j**) Shown is quantification of EdU^+^ cells in dental papilla (**i**) and dental follicle (**j**) around HERS at P7. Blue bars: Control, red bars: Osx-HDAC4 conditional KO, *n*=4 per group, *P<0.05, Mann–Whitney's *U*-test. All data are represented as mean±s.d.

We subsequently analyzed mandibular molars of HDAC4^−/−^ mice and their controls at P10 (HDAC4^−/−^ mice die around P10)^30^. The roots of the HDAC4^−/−^ mice were shorter than those seen in HDAC^+/−^ or HDAC4^+/+^ mice, with a number of prematurely embedded cementocytes appearing especially in the interradicular area (Fig. 9c, arrowheads: cementocytes). The crown morphology appeared to be comparable in all genotypes, indicating that HDAC4 is dispensable for dental crown formation. Second, we conditionally deleted HDAC4 using *Osx-cre* and a HDAC4 floxed allele^31^. *HDAC4*^fl/fl^ (Control) and *Osx-cre*; *HDAC4*^fl/fl^ (Osx-HDAC4 cKO) littermate mice were analyzed at P25. Mandibular molars of the Osx-HDAC4 conditional knockout mice exhibited significantly shorter roots than controls (Fig. 9d,e, quantification of the root length of M1D is shown in Fig. 9f, Mann–Whitney's *U*-test). The cementum of the Osx-HDAC4 conditional knockout appeared to be irregular and thicker, with occasional embedded cementocytes in the matrix (Fig. 9e, arrowheads). No obvious alteration of the crown morphology was observed in the Osx-HDAC4 conditional knockout. In addition, cell proliferation was evaluated by administering EdU to these mice shortly before analysis at P7. In the Osx-HDAC4 conditional knockout, the number of EdU^+^ cells was reduced in the dental papilla and showed a trend toward reduction in the dental follicle (Fig. 9g,h, quantification of EdU^+^ dental papilla and follicle cells is shown in Fig. 9i,j), suggesting that deletion of the HDAC4 leads to less proliferation of these mesenchymal cells.

These data suggest that deletion of HDAC4 partially recapitulated the root truncation and cementum phenotypes caused by deletion of PPR, supporting the notion that HDAC4 might be a downstream mediator of the PPR signalling in root-forming progenitors.

## Discussion

Thus far, the characteristics of mesenchymal progenitor populations for dental root-forming cells have not been fully understood. Although *Osx-creER* most likely marks heterogeneous pools of progenitor cells with distinct determined fates at the onset of root morphogenesis, at least a subpopulation of these osterix-expressing mesenchymal cells participates in subsequent root formation by providing all relevant cell types, such as odontoblasts, dental pulp cells, cementoblasts and PDL cells. In light of earlier and recent controversies related to cementogenesis and root formation, our study provides solid evidence of the mesenchymal origin of cementum and root-related tissues^32^,^33^. PPR is likely to be important for proliferation of these mesenchymal progenitors in a cell-autonomous manner. In addition, PPR is likely to orchestrate cementoblast differentiation of these progenitors, as the PPR-deficient cells failed to form the acellular cementum, and rather formed an irregular cellular cementum on the root surface. This phenotype can be interpreted as accelerated and disordered differentiation. We observed upregulation of *Nfic*, as well as tendency for an increase in the mRNA encoding the bone/cementum matrix protein, osteopontin, in PPR-deficient cells. Nfic promotes proliferation and differentiation of odontoblasts *in vitro*^34^, and its deficiency leads to truncation of molar roots^27^. How the PPR signalling regulates *Nfic* expression, and Nfic regulates cementoblast differentiation requires further investigations.

There is an increasing interest in dental root formation, and recent studies have shown roles for various signalling pathways, such as Hedgehog, BMP/TGF-β and Wnt/β-catenin pathways, in the regulation of root formation. The HERS expresses sonic hedgehog (Shh)^35^, and its expression is regulated by BMP/TGF-β/Smad4 signalling^36^. An optimal level of HERS-derived Shh is important for dental root elongation, as either deletion or activation of the hedgehog signalling in the dental mesenchyme causes shorter roots^37^. In addition, Wnt/β-catenin signalling in odontoblasts^38^ and BMP/TGF-β signalling in the dental mesenchyme^39^,^40^ are also important for tooth root formation. Understanding how these pathways influence and work with the PPR signalling will be an important agenda for the future.

We note that, as expected, “temporal conditional” deletion of *PPR* using a tamoxifen-inducible *Osx-creER* caused much milder phenotypes than conventional conditional deletion of *PPR* using a constitutively active *Osx-cre* (Figs 4 and 6), with the latter associated with unerupted molars and hypomorphic alveolar bones. The *Osx-creER* cells at P3 represent a subpopulation of the *Osx-cre*-targeted cells, as *Osx-creER* marks cells only in the lower portion of the dental follicle (Fig. 2a,b). The upper dental follicle is important for tooth eruption, while the lower dental follicle is important for root formation and alveolar bone formation^41^. The detailed mechanisms regarding how the PTHrP–PPR signalling regulates the dental follicle need further clarification. Alternatively, it is possible that the PPR signalling is important at a much earlier time during tooth development even before tooth root formation starts at P3.

The PPR haploinsufficiency is associated with primary failure of molar eruption in humans, and orthodontic intervention on these unerupted molars often results in ankylosis^42^. Whether the PPR haploinsufficiency is associated with the dental root abnormalities in humans is unknown. A possible mechanism might be that the PPR mutation leads to defective dental mesenchymal cell differentiation and partial ankylosis of molars to bone, hampering their normal eruption.

Our further study indicates that HDAC4 might be one of the major downstream mediators of the PPR signalling in dental mesenchymal progenitors. The reason for only partial recapitulation of the root phenotype might be due to redundancy of Class IIa HDACs. *Hdac4* and *Hdac5*, but not *Hdac7*, were expressed in FACS-sorted PDL cells/cementoblasts, and *Hdac5* expression level appeared to be higher than that of *Hdac4*. Therefore, it is possible that Hdac5 compensates for the loss for Hdac4 in these mice.

**Figure 10:**
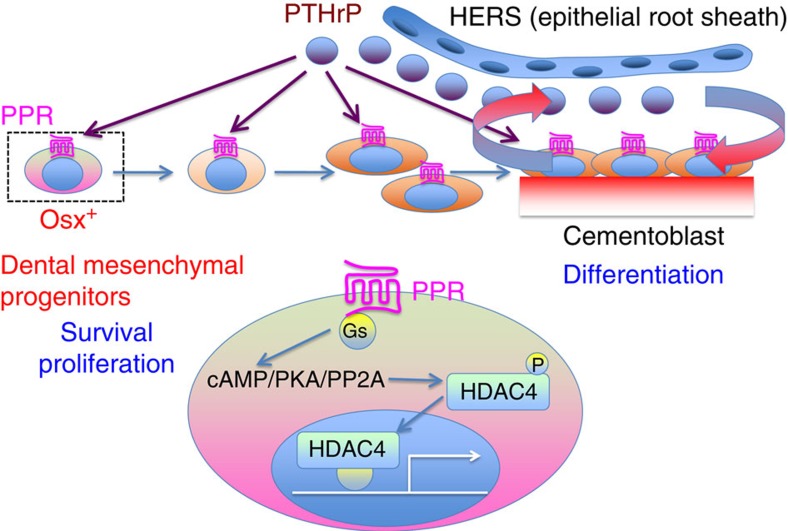
PTHrP–PPR signalling regulates cell survival, proliferation and differentiation of osterix-expressing dental mesenchymal progenitors through the HDAC4 pathway. Shown is the concluding summary diagram of this study. First, our findings suggest that PTHrP is predominantly expressed by cells in the dental follicle and root surface, where active cementogenensis takes place. Second, our findings collectively suggest that the PPR signalling in Osx^+^ dental mesenchymal progenitors is important in several critical aspects, such as cell survival, proliferation and differentiation into cementoblasts. Deletion of the PPR signalling in these progenitors leads to defects in survival, proliferation and differentiation of these cells, leading to truncated dental roots and dysregulated cementum formation. Furthermore, HDAC4 appears to be one of the major downstream mediators of the PPR signalling in dental mesenchymal progenitors.

In conclusion, our data collectively indicate that the PPR signalling in dental mesenchymal progenitors is essential for tooth root formation including orchestrated differentiation of cementoblasts, underscoring the importance of the PTHrP–PPR system in dental root formation and tooth eruption (See Summary Fig. 10).

## Methods

### Mice

The mice used in this study were described in the following references: *Osx-cre*^19^, *Oc-cre*^22^, *Dmp1-cre*^23^, *Osx-creER*^*T2*^ (ref. 8), *Col1(3.2 kb)-creER*^*T2*^ (ref. 8), *PPR-floxed*^20^, *HDAC4-floxed*^31^, *PPR*-null^43^, *PTHrP-LacZ*/null^18^, *HDAC4-LacZ*/null^30^, *Col1-caPPR*^24^, *Oc*-GFP^44^ and *Col1(2.3 kb)*-GFP^45^. *Rosa26-loxP-stop-loxP*-tdTomato^46^ (*R26R*-tomato, JAX007914) mice were acquired from Jackson laboratory. All procedures were conducted in compliance with the Guideline for the Care and Use of Laboratory Animals approved by Institutional Animal Care and Use Committee (IACUC) of the Massachusetts General Hospital and the University of Michigan. All mice were housed in a specific pathogen-free condition, and analyzed in a mixed background of C57/BL6, 129Sv and FVB/N strains. Mice were used for analysis regardless of the sex. Tail biopsies of mice were lysed by a HotShot protocol (incubating the tail sample at 95 °C for 30 min in an alkaline lysis reagent followed by neutralization) and used for PCR-based genotyping (GoTaq Green Master Mix, Promega and C1000 Touch Cycler, Bio-rad). Perinatal mice were also genotyped fluorescently (BLS miner's lamp) whenever possible. Mice were sacrificed by over-dosage of carbon dioxide or decapitation under inhalation anaesthesia in a drop jar (Aerrane isoflurane, Henry Schein). For lineage-tracing experiments, 3-days-old mice received 0.1 mg of tamoxifen intraperitoneally. Tamoxifen (Sigma T5648) was dissolved first in 100% ethanol, then in sunflower seed oil (Sigma S5007) overnight at 60 °C. Mice were genotyped by PCR and, whenever possible, also fluorescently (BLS miner's lamp).

### Histology

Mandibles were carefully dissected and fixed in 4% paraformaldehyde, overnight at 4 °C, then decalcified in 15% EDTA for 1–14 days. Decalcified samples were cryoprotected in 30% sucrose/PBS followed by 30% sucrose/PBS:optimal cutting temperature (OCT) compound (1:1) solution, each overnight at 4 °C. Samples were carefully embedded in OCT compound under a dissecting microscope (Nikon SMZ-10A) to ensure the parallelism of sectional planes, and cryosectioned at 12 μm thickness (Leica CM1850). Images were captured with a fluorescence microscope (Nikon Eclipse E800) with prefigured triple-band filter settings for DAPI/FITC/TRITC, and merged with Spot Advanced Software (Spot Imaging). Confocal images were acquired using LSM510 and Zen2009 software (Zeiss) with lasers and corresponding band-pass filters for DAPI (Ex.405 nm, BP420–480), GFP (Ex.488 nm, BP505–530), tdTomato and Alexa555 (Ex.543 nm, BP565–595) and Alexa647 (Ex.633 nm LP650). LSM Image Viewer and Adobe Photoshop software were used to capture and align images. The root length, the tooth height, cell numbers and the cementum thickness were measured using NIH Image J. The root length of the M1 distal root was defined as the distance of the root from the centre of the apex to the line connecting the cementoenamel junction and the maximum point of the root bifurcation. The tooth height of M1 in Col1-PPR* mice was defined as the distance from the centre of the apex to the centre of the enamel on the crown.

### Cell proliferation assay

To evaluate cell proliferation, EdU (Invitrogen A10044) was administered twice, 6 h and 3 h before mice were sacrificed at P7 (0.1 mg per injection) or P14 (0.2 mg per injection). Click-iT Imaging Kit (Invitrogen, C10337) with Alexa Flour 555 or 647-azide (Invitrogen A20012 or A10277) was used to detect EdU in cryosections of M1. EdU^+^ cell number was counted in the dental papilla and dental follicle surrounding HERS at P7 using NIH Image J.

### X-Gal staining of mandibular molars

Mandibular incisors were carefully removed under a dissecting microscope (Nikon SMZ-10A) to ensure the maximum penetration of the substrate. Dissected mandibles were fixed in 2% paraformaldehyde and 0.2% glutaraldehyde for 30 min at 4 °C, followed by overnight X-gal staining at 37 °C. Stained samples were further postfixed in 4% paraformaldehyde, overnight at 4 °C, then decalcified in 15% EDTA for 1–14 days. Decalcified samples were cryoprotected in 30% sucrose/PBS followed by 30% sucrose/PBS:OCT (1:1) solution, each overnight at 4 °C.

### Immunohistochemisty

Cryosections were postfixed in 4% paraformaldehyde for 15 min, blocked with 2% BSA/TBST for 30 min and incubated with rabbit anti-GFP polyclonal antibody (1:200, Abcam ab290) or rabbit anti-periostin polyclonal antibody (1:2,000, Millipore ABT280) overnight at 4 °C, and subsequently with Alexa Fluor 488-conjugated goat anti-rabbit IgG (Invitrogen A11034) or Alexa Fluor 546-conjugated goat anti-rabbit IgG (Invitrogen A11035) for 3 h at 4 °C. Sections were further incubated with DAPI (4′,6-diamidino-2-phenylindole) to stain nuclei. To immunohistochemically detect PPR, freshly isolated P7 mandibles were embedded in super cryosection embedding medium (Section-Lab, Hiroshima, Japan) on dry ice. Sections of mandibles were cut without decalcification using a tungsten blade and a tape transfer system (Cryofilm IIC; Section-Lab). Non-decalcified sections were adhered on the adhesive side of the cryofilm and analyzed as attached throughout. Sections on cryofilms were dried completely, postfixed in 4% paraformaldehyde for 15 min, blocked with 3% BSA/TBST for 30 min and incubated with rabbit anti-PPR polyclonal antibody (1:200, AssaybioTech G220) overnight at 4 °C, and subsequently with Alexa Fluor 546-conjugated goat anti-rabbit IgG (Invitrogen A11035) for 3 h at 4 °C. Sections were further incubated with DAPI to stain nuclei.

### FACS and quantitative real-time PCR analysis

Maxillary and mandibular first and second molars (M1 and M2) of P25 mice were carefully elevated and extracted^26^. Briefly, after gingiva was curettaged with dental spatula No. 7, molars were carefully removed from the sockets using the extraction forceps specifically designed for rodent molars (Sanshou, Tokyo, Japan) under a dissection microscope (Nikon SMZ-10A). Only molars without root fracture were used for subsequent digestion. Extracted molars were incubated twice with two Wunsch units of Liberase TM (Roche) in Ca^2+^, Mg^2+^-free HBSS (Sigma) at 37 °C for 45 min on a shaking incubator (Thermomixer, Eppendorf). The supernatant was carefully filtered through a cell strainer, and harvested cells were stained for anti-mouse CD45-APC (1:500, eBioscicence) in DPBS/2%FBS on ice for 30 min. Cell sorting was performed using a five-laser BD FACS Aria II (Ex.355/407/488/532/633 nm) and FACSDiva, and cells were directly sorted into TRIzol LS reagent (Invitrogen). Total RNA was isolated using NucleoSpin RNA XS kit (Macherey-Nagel) with a carrier RNA. First-strand cDNA was synthesized using QuantiTect Reverse Transcription kit (Qiagen). Quantitative real-time PCR analysis was carried out using QuantiFast SYBR Green PCR kit (Qiagen) and StepOne Plus Real-time PCR systems (Applied Biosystems). The PCR conditions were 95 °C for 10 s and 60 °C for 30 s for 50 cycles. The primer sequences are as follows: *Actb*, 5′-GGCTGTATTCCCCTCCATCG-3′ (forward) and 5′-CCAGTTGGTAACAATGCCATGT-3′ (reverse); *PPR (E1)*, 5′-GCTGCTCAAGGAAGTTCTGC-3′ (forward) and 5′-CGTCCACCCTTTGTCTGACT-3′ (reverse); *Nfic*, 5′-CATCGCGGTACACAGTGG-3′ (forward) and 5′-GGCCGTATGGGGGAAGTA-3′ (reverse); *Col1a1*, 5′-AGACATGTTCAGCTTTGTGGAC-3′ (forward) and 5′-GCAGCTGACTTCAGGGATG-3′ (reverse); *Bsp*, 5′-GAAAATGGAGACGGCGATAG-3′ (forward) and 5′-CATTGTTTTCCTCTTCGTTTGA-3′ (reverse); *Opn*, 5′-CCCGGTGAAAGTGACTGATT-3′ (forward) and 5′-ATCTGGGTGCAGGCTGTAA-3′ (reverse); *Dmp1*, 5′-GGTTTTGACCTTGTGGGAAA-3′ (forward) and 5′-CATATTGGGATGCGATTCCT-3′ (reverse); *Hdac4*, 5′-AATCCTGCCCGTGTGAAC-3′ (forward) and 5′-GTAGGGGCCACTTGCAGA-3′ (reverse); *Hdac5*, 5′-GAGTCCAGTGCTGGTTACAAAA-3′ (forward) and 5′-GTACACCTGGAGGGGCTGT-3′ (reverse); *Hdac7*, 5′-CGCCAGTTGGAAACAATGAT-3′ (forward) and 5′-GCTGAGAGCCTGGTGTGTCT-3′ (reverse).

### 3D microCT analysis

Mandibles including molars and incisors were scanned at a thickness of 3 μm per section using Scanco μCT 100 microCT system.

### Statistical analysis

Results were represented as mean values±s.d. Statistical evaluation was conducted on the basis of One-way ANOVA followed by a post-hoc test or Mann–Whitney's *U*-test. A *P* value of <0.05 was considered significant. No statistical method was used to pre-determine sample size. Sample size was determined on the basis of previous literature and our prior experience that give sufficient s.d. of the mean so as not to miss a biologically important difference between groups. The experiments were not randomized. All the available mice of the desired genotypes were used for experiments. The investigators were not blinded to during experiments and outcome assessment. One mandible from each mouse was arbitrarily chosen for histological analysis. Genotypes were not particularly highlighted during quantification.

## Additional information

**How to cite this article:** Ono, W. *et al*. Parathyroid hormone receptor signalling in osterix-expressing mesenchymal progenitors is essential for tooth root formation. *Nat. Commun.* 7:11277 doi: 10.1038/ncomms11277 (2016).

## Supplementary Material

Supplementary Information Supplementary Figures 1-5

## Figures and Tables

**Figure 1:**
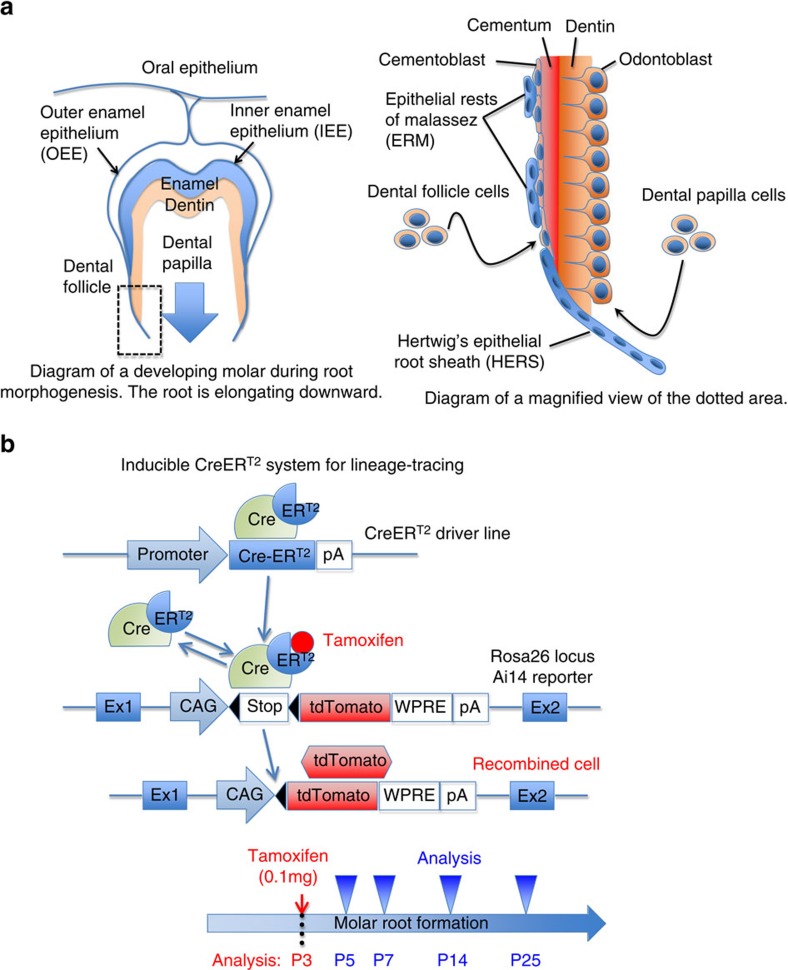
A lineage-tracing model to study tooth root formation. (**a**) Shown is the diagram of a developing molar during root morphogenesis. Enamel is produced by ameloblasts derived from inner enamel epithelium (IEE). Dentin is produced by odontoblasts derived from dental papilla mesenchyme. Cementum is produced by cementoblasts derived from dental follicle mesenchyme. HERS is formed by fusion of outer and inner enamel epithelia, and becomes a physical demarcation between dental papilla and follicle. HERS eventually breaks down and becomes epithelial rests of Malassez (ERM). (**b**) Shown is the diagram of a tamoxifen-inducible creER^T2^ system for lineage tracing. CreER^T2^ recombinase is expressed by a named promoter. It excises the stop codons in the Rosa26 locus only in the presence of tamoxifen. Once the stop codons are removed, the targeted cells permanently express tdTomato in a ubiquitously active CAG promoter-dependent manner. A low-dose tamoxifen (0.1 mg) is administered intraperitoneally, when murine molar root morphogenesis starts around postnatal day 3 (P3). Tamoxifen stays active upto 48 h after injection.
