# Supplementary material for: Parathyroid hormone receptor signalling in osterix-expressing mesenchymal progenitors is essential for tooth root formation
Source: Nat Commun. 2016 Apr 12;7:11277. doi: 10.1038/ncomms11277 (PMC4832076; doi:10.1038/ncomms11277)
Supplement: Supplementary Information — Supplementary Figures 1-5 [file ncomms11277-s1.pdf]

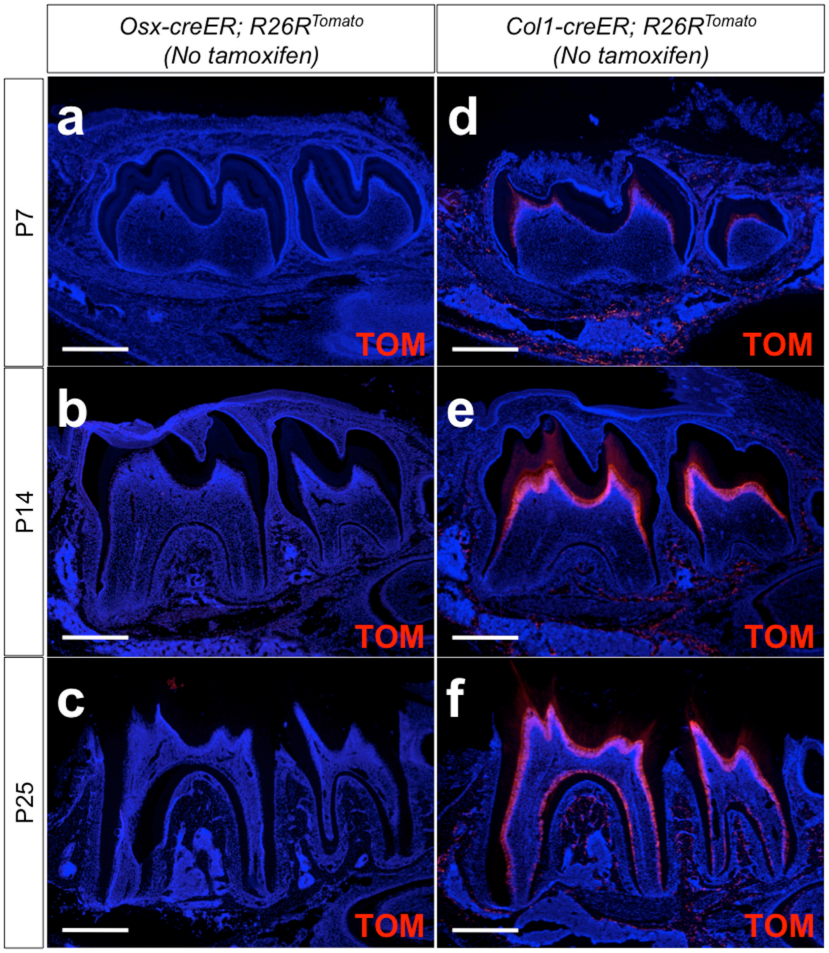

Supplementary Figure 1, related to Figure 2. Mesenchymal progenitors of dental root-forming cells express osterix (Osx).

a-c. *Osx-creER; R26R*^{Tomato} mice without tamoxifen injection were analyzed at P7 (a), P14 (b) and P25 (c). M1 sections were stained for nuclei. x100, red: tdTomato, blue: DAPI.

d-f. *Col1-creER; R26R*^{Tomato} mice without tamoxifen injection were analyzed at P7 (d), P14 (e) and P25 (f). M1 sections were stained for nuclei. x100, red: tdTomato, blue: DAPI. Scale bars, 500μm.

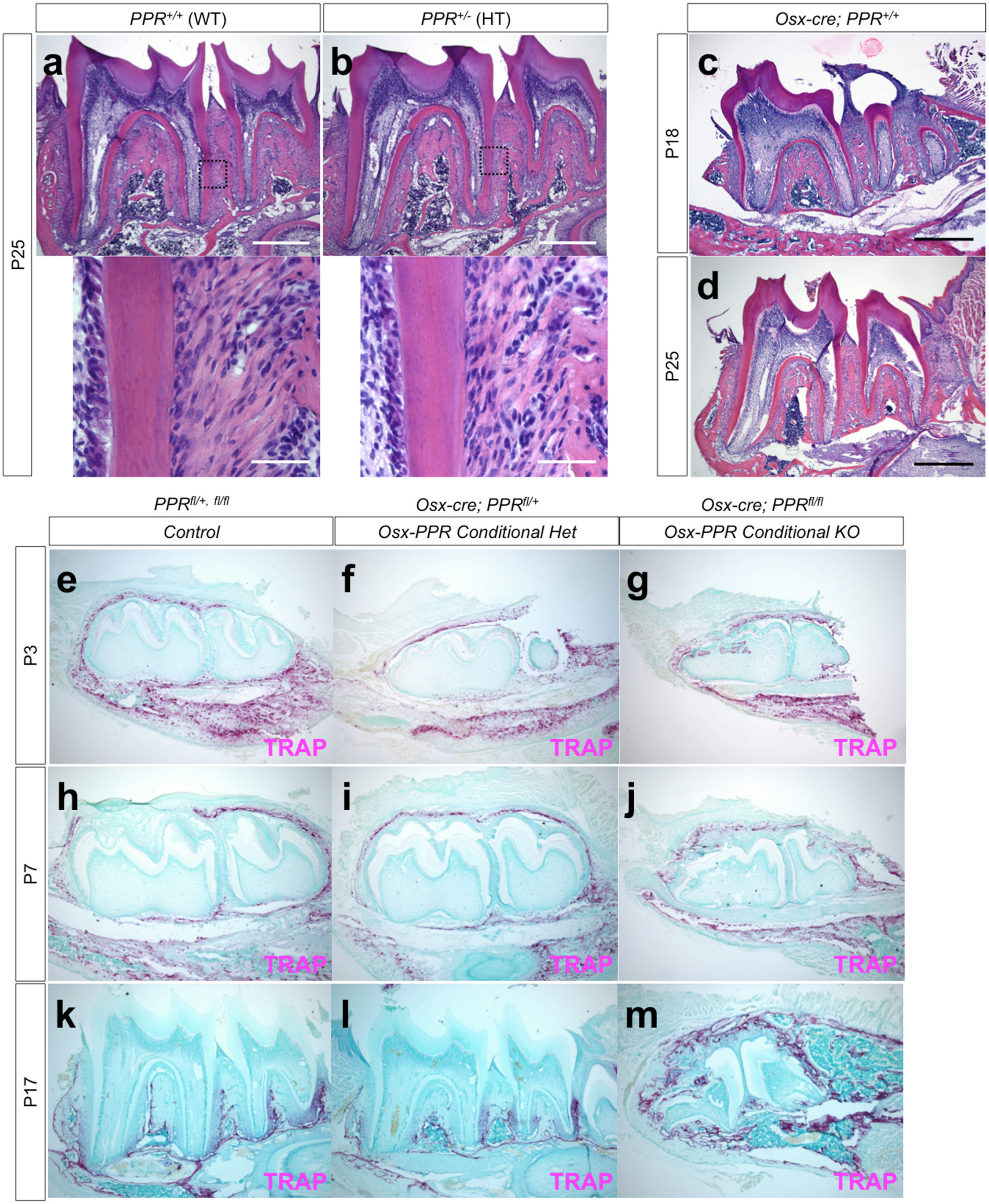

Supplementary Figure 2, related to Figure 4. Tooth root formation requires PTH/PTHrP receptor in osterix-lineage cells.

a,b. Mandibular first molar (M1) sections of (a) *PPR*$^{+/+}$ (WT) and (b) *PPR*$^{+/-}$ (HT) at P25 were stained for H&E. Lower panels show a magnified view of the dotted area. Scale bars represent 500µm (upper panels) and 50µm (lower panels).

c,d. M1 sections of *Osx-cre*; *PPR*$^{+/+}$ at P18 (c) and P25 (d) were stained for H&E.

e-m. M1 sections of Control (e,h,k), Osx-PPR conditional Het (f,i,l) and Osx-PPR conditional KO (g,j,m) stained for TRAP activities. Sections at P3 (e,f,g), P7 (h,i,j) and P17 (k,l,m) are shown.

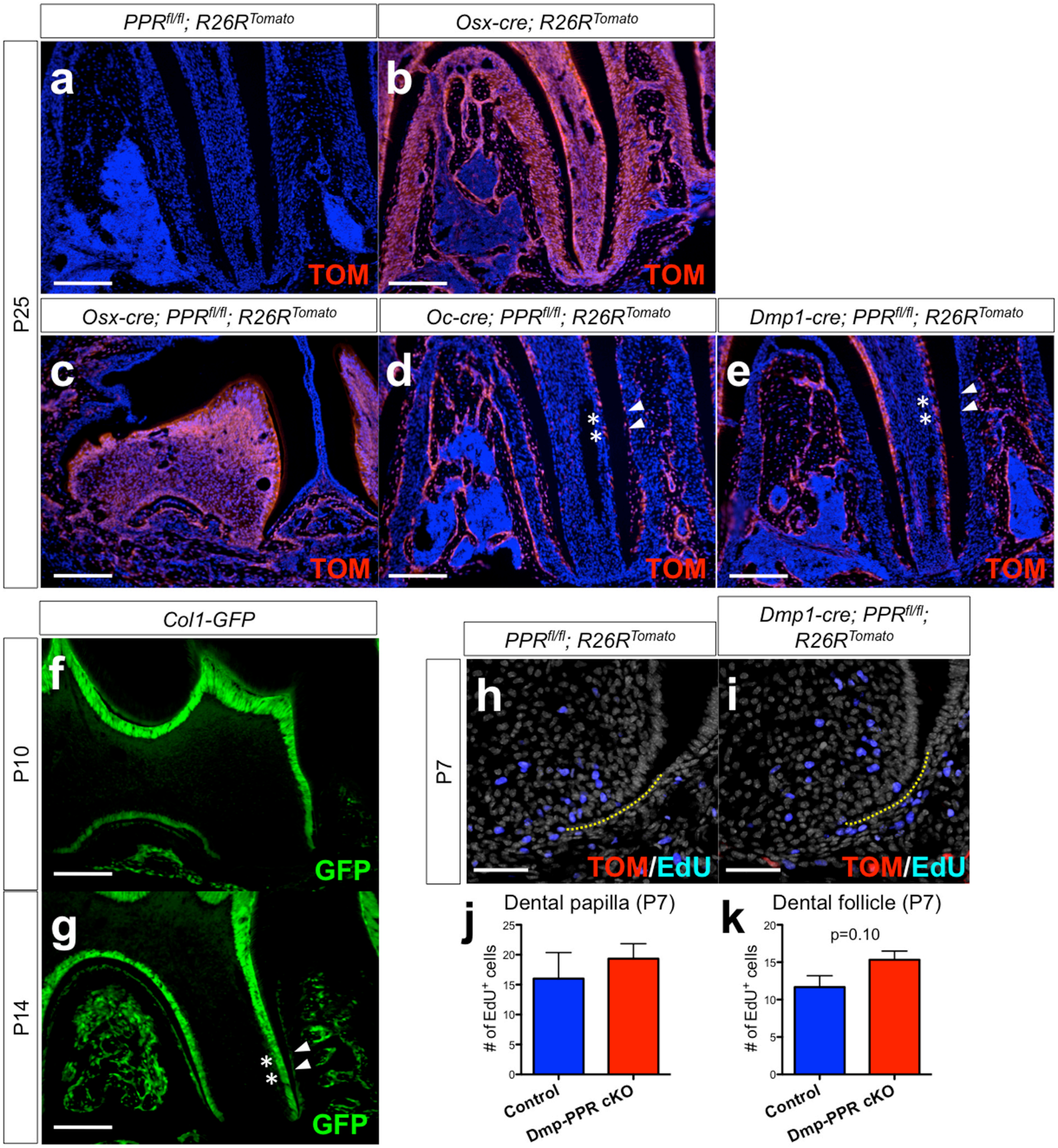

Supplementary Figure 3, related to Figure 6. PTH/PTHrP receptor in differentiated matrix-producing cells is dispensable for dental root formation

a-e. P25 mandibular first molar (M1) sections of (a) *PPR*$^{fl/fl}$; *R26R*^{Tomato}, (b) *Osx-cre*; *R26R*^{Tomato}, (c) *Osx-cre*; *PPR*$^{fl/fl}$; *R26R*^{Tomato}, (d) *Oc-cre*; *PPR*$^{fl/fl}$; *R26R*^{Tomato} and (e) *Dmp1-cre*; *PPR*$^{fl/fl}$; *R26R*^{Tomato} were stained for nuclei. Red represents tdTomato and blue represents DAPI. Asterisks indicate odontoblasts and arrowheads point to cementoblasts. Scale bars represent 200μm

f,g. Shown are *Col1(2.3kb)*-GFP M1 sections at P10 (f) and P14 (g). Green represents EGFP. Asterisks indicate odontoblasts, arrowheads point to cementoblasts. Scale bars represent 200μm.

h,i. Sections of (h) Control (*PPR*$^{fl/fl}$; *R26R*^{Tomato}) and (i) Dmp1-PPR conditional KO (*Dmp1-cre*; *PPR*$^{fl/fl}$; *R26R*^{Tomato}) mice at P7 were stained for nuclei and EdU. EdU was administered twice (6 and 3 hours) prior to analysis. Red represents tdTomato, blue represents EdU-Alexa647 and gray represents DAPI. Yellow dotted lines highlight HERS. Scale bars represent 50μm.

j.k. Shown is quantification of EdU^{+} cells in dental papilla (p) and dental follicle (q) around HERS at P7. Blue bars represent Control and red bars represent Dmp1-PPR conditional KO, n=3 per group.

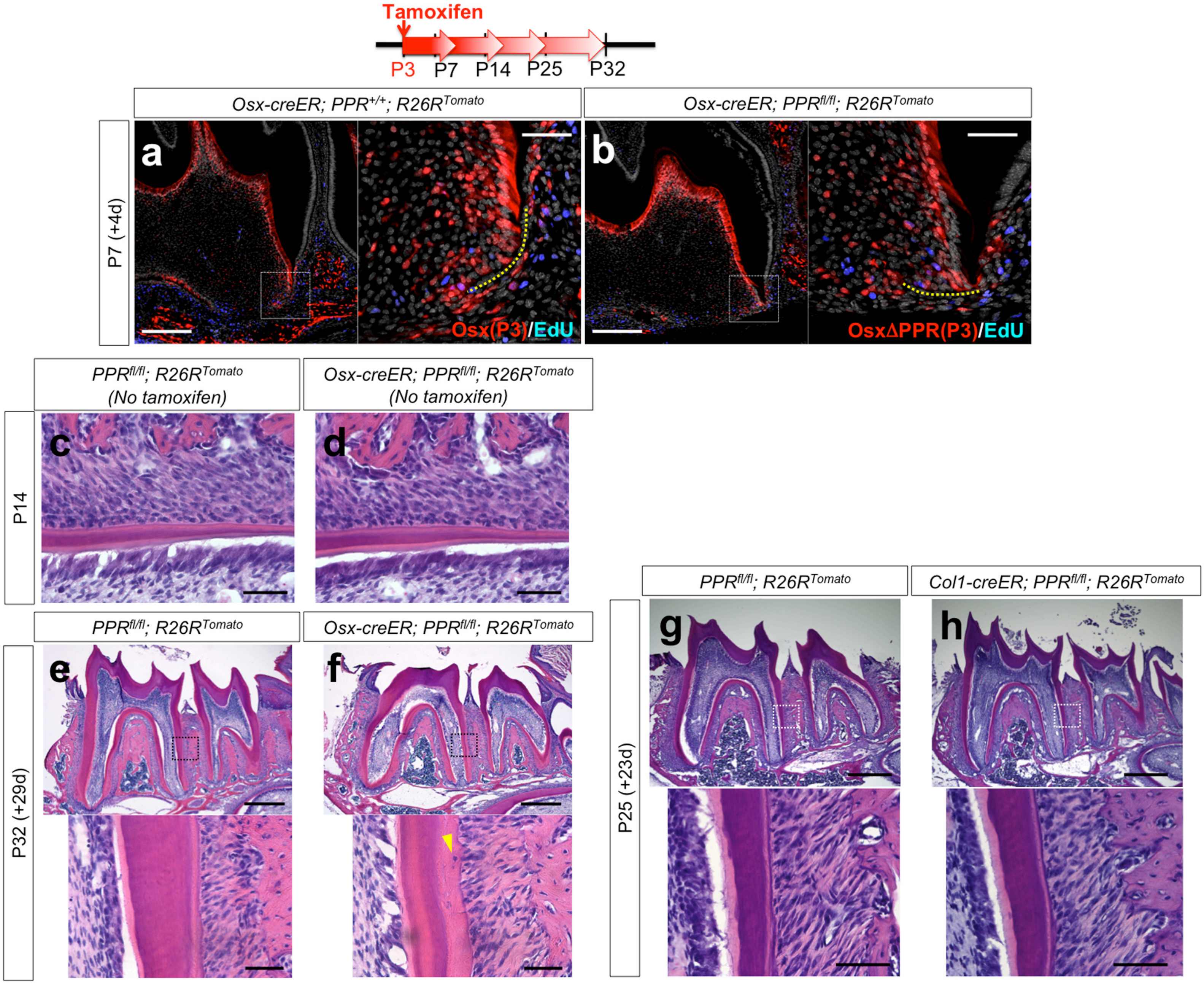

Supplementary Figure 4, related to Figure 7. PTH/PTHrP receptor orchestrates cementoblast differentiation of osterix-expressing progenitors.

a,b. *Osx-creER; PPR$^{+/+}$; R26R^{Tomato}* (a) and *Osx-creER; PPR$^{fl/fl}$; R26R^{Tomato}* (b) mice received tamoxifen at P3 and were analyzed at P7. EdU was administered twice (6 and 3 hours) prior to analysis. Mandibular first molar (M1) sections were stained for nuclei and EdU. Right panel shows a magnified view of the dotted areas. Blue represents EdU-Alexa647, red represents tdTomato and gray represents DAPI. Yellow dotted lines highlight HERS. Scale bars represent 200µm (left panel) and 50µm (right panel).

c,d. M1 sections of (c) *PPR$^{fl/fl}$; R26R^{Tomato} and (d) Osx-creER; R26R^{Tomato}* molars without tamoxifen injection at P14 were stained for H&E. Scale bars represent 50µm.

e.f. *PPR$^{fl/fl}$; R26R^{Tomato}* (e) and *Osx-creER; PPR$^{fl/fl}$; R26R^{Tomato}* (f) mice received tamoxifen at P3 and were analyzed at P32. Mandibular first molar (M1) sections were stained for H&E. Lower panels show a magnified view of the dotted areas. Yellow arrowhead points to embedded cementocyte. Scale bars represent 500µm (upper panels) and 50µm (lower panels).

g.h. *PPR$^{fl/fl}$; R26R^{Tomato}* (e) and *Col1-creER; PPR$^{fl/fl}$; R26R^{Tomato}* (f) mice received tamoxifen at P3 and were analyzed at P25. Mandibular first molar (M1) sections were stained for H&E. Lower panels show a magnified view of the dotted areas. Scale bars represent 500µm (upper panels) and 50µm (lower panels).

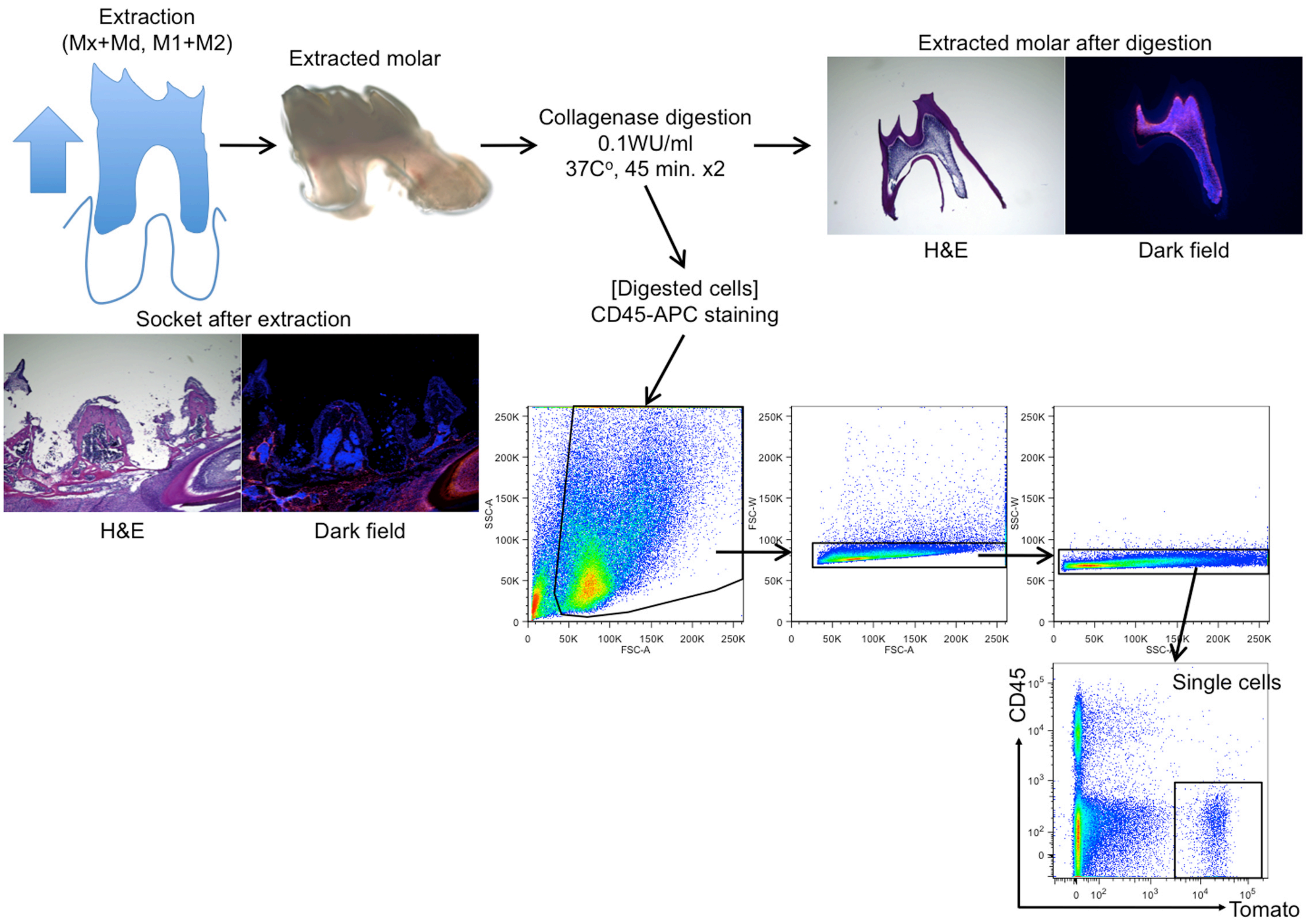

Supplementary Figure 5. related to Figure 7. PTH/PTHrP receptor orchestrates cementoblast differentiation of osterix-expressing progenitors.

Shown is the diagram of FACS analysis and sorting for collagenase-digested periodontal ligament cells/ cementoblasts is shown. Maxillary and mandibular first and second molars (M1 and M2) of P25 mice were carefully extracted under surgical loupes using specially designed extraction forceps. Extracted molars were subjected to two rounds of collagenase digestion. The socket after extraction showed a great majority of periodontal ligament cells were attached to extracted molars (lower left panel). The collagenase-digested molars showed no cell left on the root surface, while the dental pulp was intact and undigested (upper right panel). Digested cells were stained for CD45-APC, and gated on FSC/SSC to analyze single cells.
